# miR-30b-5p inhibits proliferation, invasion, and migration of papillary thyroid cancer by targeting GALNT7 via the EGFR/PI3K/AKT pathway

**DOI:** 10.1186/s12935-021-02323-x

**Published:** 2021-11-24

**Authors:** Ye Wang, Congjun Wang, Zhao Fu, Siwen Zhang, Junqiang Chen

**Affiliations:** 1 The First Affiliated Hospital of Guangxi Medical University, Department of Gastrointestinal Gland Surgery, Nanning, 530021 Guangxi China; 2 Guangxi Medical University, Guangxi Key Laboratory of Enhanced Recovery after Surgery for Gastrointestinal Cancer, Nanning, 530021 Guangxi China

**Keywords:** Papillary thyroid carcinoma, miR-30b-5p, GALNT7, The EGFR/PI3K/AKT pathway, Progression

## Abstract

**Background:**

Papillary thyroid carcinoma (PTC) is a common endocrine tumor. Increasing evidence has shown that microRNA dysfunction is involved in the occurrence and development of cancer. The expression of MicroRNA-30b-5p (miR-30b-5p) was down-regulated in PTC; however, its role in the development of PTC is not clear. Hence, this study aimed to explore the role and mechanism of miR-30b-5p in the occurrence and development of PTC.

**Methods:**

The qRT-PCR assay was used to detect the expression of miR-30b-5p in 60 cases of papillary thyroid carcinoma along with their matched non-cancerous tissues. This study explored the biological function of miR-30b-5p by the functional gain and loss experiments in vitro and vivo. The direct target gene of miR-30b-5p and its signaling pathway was identified through bioinformatics analysis, qRT-PCR, western blot, rescue experiments, and double luciferase 3'-UTR report analysis.

**Results:**

This study demonstrated that the low expression of miR-30b-5p is related to poor clinicopathological features. Functionally, the overexpression of miR-30b-5p inhibited the proliferation, invasion, and migration of PTC cells. Bioinformatics and luciferase analysis showed that GALNT7 is the direct and functional target of miR-30b-5p. Moreover, miR-30b-5p inhibited the proliferation of PTC in vivo by inhibiting the expression of GALNT7. The studies on the mechanism have shown that GALNT7 promotes cell proliferation and invasion by activating EGFR/PI3K/AKT kinase pathway, which can be attenuated by the kinase inhibitors.

**Conclusions:**

Overall, miR-30b-5p inhibited the progression of papillary thyroid carcinoma by targeting GALNT7 and inhibiting the EGFR/PI3K/AKT pathway.

**Supplementary Information:**

The online version contains supplementary material available at 10.1186/s12935-021-02323-x.

## Background

Thyroid cancer is a common endocrine malignancy, and its incidence has shown a rapid increase in recent decades [1]. Differentiated thyroid cancer (DTC) accounts for more than 90% of all thyroid cancers, including the papillary, follicular, and poorly differentiated histological subtypes [2]. Although most patients diagnosed with DTC have a good prognosis, a significant proportion of patients have persistent or recurrent disease, with a minority reporting death [3]. Its occurrence and development seem as complex as a network system, and its underlying mechanism is still largely unknown. Thus, it is critical to explore the underlying molecular mechanisms and identify the potential therapeutic targets in papillary thyroid carcinoma (PTC).

Mature microRNAs (miRNAs) belong to a class of evolutionally conserved, single-stranded, small (approximately 19–23 nucleotides), endogenously expressed, and non-protein-coding RNAs that act as post-transcriptional regulators of gene expression in a wide variety of animals, plants, and viruses [4]. Numerous studies have documented the role of miRNA in cancer progression [5], and several miRNAs have been reported to be associated with thyroid carcinomas [6–10]. Overexpression of miR-146b-5p and miR-146b-3p is associated with PTC metastasis [9]. MiR-139-5p can significantly reduce the migration and proliferation of anaplastic thyroid cancer cells [6]. MiR-125a-5p exerts its tumor inhibitory effect by directly inhibiting the expression of CD147 protein in thyroid cancer [7]. MiR-222 promotes the invasion and metastasis of thyroid cancer by targeting PPP2R2A [8]. Additionally, the miR-30b-5p is reported as a new diagnostic marker to improve the diagnosis of PTC [11]. MiR-30b-5p often acts as a tumor suppressor, inhibiting the proliferation and metastasis of cancer, including renal cell carcinoma [12], lung cancer [13], and colorectal cancer [14]. In terms of mechanism, miR-30b-5p is reported to control tumor or disease progression by regulating autophagy [15–18], epithelial-mesenchymal transition (EMT) [12, 19], and mTOR signaling pathway [20]. However, the role of miR-30b-5p in PTC is not very clear.

Overexpression of epidermal growth factor receptor (EGFR) is associated with thyroid carcinoma progression [21]. Recent studies have found that the detection of EGFR expression in patients with PTC is helpful to judge the prognosis of patients with PTC [22–25]. In addition, EGF/EGFR signal pathway plays a crucial role in the EMT progression of thyroid cancer cells [26, 27]. EGFR is activated by its ligand epidermal growth factor (EGF), which triggers a signal cascade, resulting in enhanced migration and invasion of thyroid cancer cells [28]. There is no doubt that the expression level of EGFR is also regulated by miRNA. MiR-146b blocks the EGFR pathway to inhibit the progression of ovarian cancer [29]. MiR-137 inhibits growth and invasion by targeting EGFR in thyroid cancer cells [30]. MiR-7 affects the progress of PTC by regulating the expression of EGFR [31]. However, the relationship between MiR-30b-5p and EGFR is not clear.

Our results showed that the expression of miR-30b-5p in PTC was significantly lower than that of the paracancerous tissues. MiR-30b-5p plays the role of the tumor suppressor gene by directly inhibiting the expression of Polypeptide *N*-Acetylgalactosaminyltransferase 7(GALNT7). The abnormal expression of GALNT7 and its family members is related to the occurrence and development of many kinds of malignant tumors [32–40]. Therefore, we focused on the interaction between MiR-30b-5p and GALNT7 in PTC cells. We report a new regulatory pathway composed of MiR-30b-5p/GALNT7/EGFR, which provides potential biomarkers and therapeutic targets for the diagnosis and treatment of PTC.

## Methods

### Patients and samples

The PTC and non-tumor tissues were collected from 60 PTC patients admitted to the first affiliate Hospital of Guangxi Medical University in the past year (Additional file 2: Table S1). They were initially diagnosed with PTC and received PTC lobectomy. Tumor tissues were immediately collected, and the non-tumor tissues were sampled more than 5 cm away from the tumor border. The samples were stored using an RNA protective reagent (RNA isolater Total RNA Extraction Reagent, R401-01, Vazyme) at – 80 °C. None of these patients received any chemotherapy or radiotherapy before the operation. This study and its informed consent were reviewed and certified by the Ethics Committee of the first affiliated Medical College of Guangxi Medical University [2015 (KY-E-018)].

### Cell culture and transfection

PTC cell lines TPC-1, KTC-1, and B-CPAP were purchased from Wuhan Pu-nuo-sai Life Technology Co. Ltd. (Wuhan, China). K-1 was purchased from Shanghai Gene Chemical Technology Co., Ltd. Normal thyroid epithelial cell line, namely, Nthy-ori-3-1, were purchased from Shanghai Zhongqiao Xinzhou Biotechnology Co., Ltd. Cells were recently authenticated by STR profiling. Cells are routinely tested for mycoplasma prior to experimentation (MycoAlert Detection Kit (#LT07-418, Lonza). All cell lines were cultured in RPMI 1640 with 10% fetal bovine serum (FBS, Gibco-BRI, USA) and incubated at 37 °C with 5% CO_2_. Cells were digested using 0.25% trypsin and EDTA. MiR-30b-5p (agomir, mimics, and inhibitor), NC (Negative Control-agomir, -mimics, and -inhibitor), si-GALNT7, and si-NC were designed by Guangzhou Ribobio Co., Ltd. GALNT7 overexpressed plasmids and corresponding negative control groups were constructed by Shanghai Genechemical Technology Co., Ltd. The transfection was performed when cell confluency reached approximately 80–90%. The miRNA mimic, miRNA inhibitor, small interference RNAs(siRNAs), and plasmids were transfected using Lipofectamine 3000 transfection reagent (Invitrogen) according to the manufacturer’s protocol. The culture medium was replaced 8 h post-transfection. Subsequent experiments were carried out after 48 h.

### Western blotting

Total protein was extracted by the lysis method using RIPA and 1% PMSF for 30 min at 4 °C and then centrifuged at 12,000 rpm for 15 min at 4 °C. The supernatant liquid was transferred to another EP tube and boiled in 5× SDS loading buffer at 95 °C for 5 min. Before boiling, the concentration of the total protein was estimated by the BCA assay. The amount of the loaded protein per lane was 30 μg. The proteins were electrophoresed in 10% SDS-PAGE gels and transferred to PVDF membranes. The membranes were incubated with 5% bovine serum albumin (BSA) in TBST for 1 h at room temperature and then incubated overnight at 4 °C with the primary antibodies. The following day, membranes were incubated with secondary antibody for 1 h. Finally, the proteins were detected by incubating the membranes with a chemiluminescent substrate (Pierce™ ECL Western Blotting Substrate, Thermo Scientific, # 32106) and were exposed subsequently to the FlourchemE Imaging System. The used primary antibodies and inhibitors are listed as follows: GALNT7 rabbit Polyclonal Antibody (Thermo Fisher Scientific, # PA5–64163, 1:1000); E-cadherin rabbit Polyclonal antibody (Proteintech, #20874–1-AP, 1:10,000); Vimentin mouse Monoclonal antibody (Proteintech, #60330–1-Ig, 1:20,000); SNAI1 rabbit Polyclonal antibody (Proteintech, #13099–1-AP, 1:500); EGFR Rabbit Monoclonal antibody (Abcam, # ab52894, 1:1000); PI3K Rabbit Monoclonal antibody (CST, #4249, 1:1000); Phospho-PI3K p85 (Tyr458)/p55 (Tyr199) antibody (CST, #4228, 1:1000); AKT rabbit Polyclonal antibody (Proteintech, #10176–2-AP, 1:1000); Phospho-AKT (Ser473) mouse Monoclonal antibody (Proteintech, #66444–1-Ig, 1:2000); GAPDH mouse Monoclonal antibody (Proteintech, #60004–1-Ig, 1:10,000); BMS-599626 (AC480) (Abcam, #ab142063): BMS-599626 was diluted in DMSO (10uM) and added after 24 h of cell culture (the volume of DMSO was less than 1%). The follow-up experiment was carried out after 72 h of culture.

### Real-time quantitative PCR

RNA samples from the frozen thyroid tissue specimens and cultured cells were extracted using the FastPure® Cell/Tissue Total RNA Isolation Kit V2 (Vazyme, Nanjing, China). The concentration and purification of RNA were detected by the Nanodrop 2000 Spectrophotometer (Thermo Scientific, USA). The concentration was above 1000 ng/ul, and A260/A280 ratio was between 1.8 and 2.0. The cDNA was generated according to the manufacturer’s instructions (Vazyme HiScript III RT SuperMix for qPCR (+ gDNA wiper), Cat# R323-01and Takara Mir-X miRNA First-Strand Synthesis Kit, Cat# 63813). The resulting cDNA was used in real-time PCR detection, which was performed by the 7500 real-time PCR system (Applied Biosystems, Waltham, MA, USA) using the ChamQTM Universal SYBR® qPCR Master Mix and miRNA Universal SYBR qPCR Master Mix (Vazyme). The qPCR conditions were set according to the manufacturer’s instructions (The conditions for the qPCR were shown in S1). All the primers were synthesized by Takara Biotechnology Co., Ltd. (Takara, China). MiR-30b-5p and GALNT7 expression was calculated as 2^ ^(−ΔΔCT)^, which was normalized using U6 and GAPDH expression, respectively. Primers used were as follows: GALNT7 forward: 5′-TCCTCGGTAACTTTGAACCCA-3′, GALNT7 reverse: 5′-GCGGTCCAGTGAGATCATGTC-3; miR-30b-5p forward: 5′-CCGAAACATCCTACACTCAGCTAA -3′, miR-30b-5p reverse: 5′-CAGTGCGTGTCGTGGAGT-3′; U6 forward: 5′-CTCGCTTCGGCAGCACA -3′, U6 reverse: 5′-AACGCTTCACGAATTTGCGT -3′; GAPDH forward: 5′-TGTGGGCATCAATGG ATTTGG -3′, GAPDH reverse: 5′-ACACCATGTATTCCGGGTCAAT -3′

### Analysis of THCA data and prediction of downstream target genes of miRNA

The TCGA (The Cancer Genome Atlas) data of 573 patients with thyroid carcinoma were downloaded from the TCGA-GDC website (https://portal.gdc.cancer.gov, last accessed March 20, 2021). The enrichment analysis of the GO (Gene Ontology) and KEGG (Kyoto Encyclopedia of Genes and Genomes) pathway was performed using the GSEA software (Gene set enrichment analysis, GSEA v. 4.0). The differential expression of mir-30b-5p was tested using the package limma. TargetScan (http://www.targetscan.org), miRDB (http://mirdb.org/miRDB), miRmap (https://mirmap.ezlab.org/), PITA (http://genie.weizmann.ac.il/pubs/mir07/mir07_data.html), and miRanda (http://www.microrna.org) websites were used to explore the downstream targets of miR-30b-5p.

### CCK-8 assay and EdU incorporation assay

Cell proliferation was monitored using the CCK-8 kit (Dojindo, USA). A day before transfection, cells were seeded into 96-well culture plates at a density of 2 × 10^3^ cells/well. The number of proliferating cells was determined by the absorbance measured at 450 nm using a microplate reader (96-well microplate, Corning) at 0, 24, 48, and 72 h. All experiments were repeated thrice. The EdU incorporation assay was performed using a BeyoClick™ EdU cell proliferation kit with Alexa Fluor 567 (RiboBio, Guangzhou, China). The treated PTC cells were cultured in 96-well plates for 24 h. Then, 100 µL of 50 µM EdU culture medium was added to each well and incubated for 2 h. Each well was further incubated with 4% polyformaldehyde at room temperature for 30 min. Later, 1X Apollo and DAPI staining reagents were added sequentially and incubated at room temperature in a decolorizing shaker for 30 min. The images were captured using an Olympus IX81 inverted fluorescent microscope and CellSens software (Olympus Corporation). All experiments were repeated thrice.

### Wound-healing assay

The treated cells (K-1 and B-CPAP) were seeded into 6-well plates for overnight culture. Before scratching, the cells were starved for 24 h in a medium containing 0% fetal bovine serum (FBS). A wound of similar size was introduced into a monolayer with a 200 µL pipette tip. Wounded monolayer cells were washed thrice with PBS to remove cell debris and then cultured further. After 24 h, the speed of wound closure was monitored and photographed. All the experiments were performed in triplicates.

### Transwell assay

Transwell invasion and migration assay was performed using 8.0 µm Transwell Permeable Support (Corning). The transfected PTC cells were cultured in serum-free RPMI 1640 medium for 24 h at 37 ℃ and then suspended in the same medium. The cells were condensed to 3 × 10^4^ cells in 200 µL cell suspension and then added to the upper chamber for migration test or Matrigel-coated upper chamber for invasion experiment. The RPMI 1640 medium with 10% FBS was added to the bottom chamber. After 24 h of incubation, the filter membrane was fixed with 90% ethanol and stained with crystal violet. For visualization, five random areas of each chamber were counted using an inverted microscope (Olympus Corporation).

### Flow cytometry CFSE assay

For apoptosis assay, the Annexin V-FITC/PI apoptosis kit (MULTI SCIENCES, Hangzhou, China) was used, and the procedure was followed as per the manufacturer’s protocols. Firstly, cells (K-1 and B-CPAP) were harvested and washed twice using pre-cold PBS buffer. Then, 5 µL of PI Staining Solution and 5 µL of Annexin V-FITC were added to each sample, and cells were incubated for 10 min. BD FACSVerse flow cytometer was used to measure the cell apoptosis. The data of the cell cycle and apoptosis assay were analyzed by BD FlowJo™ v10.7.

### Luciferase reporter assay

The wild-type GALNT7 or mut-GALNT7 fragment was constructed and inserted downstream of the pmiR-RB-Report™ (Ribobio, China) luciferase reporter gene. 293 T cells were transfected with 50 ng of reporter construct and 50 nM of miRNA mimic per well using Lipofectamine 3000 (Invitrogen, # L3000–015). The culture medium was changed 6 h after transfection. After 48 h, the cells were lysed using the passive lytic buffer (Promega, #E1910), and the reporter gene expression was detected by the dual-luciferase reporter analysis system (Promega, #E1910). All transfection experiments were performed in triplicates.

### Immunohistochemistry (IHC) and immunofluorescence (IF)

Thyroid cancer tissue was fixed with 10% formalin, and then the paraffin-embedded sections were treated with specific primary antibodies. After incubating overnight at 4 °C, the slices were washed twice and further incubated with SignalStain® Boost IHC detection reagent (CST, # 8114) at room temperature. Then, slices were labeled using SignalStain DAB substrate kit (CST, #8059) and observed under an inverted microscope (Olympus Corporation). The experiment was repeated at least thrice. For IF analysis, the cell climbing slices were incubated overnight with anti-E-cadherin and anti-vimentin antibodies at 4 °C. After three washing steps in PBS, cells were incubated in secondary antibodies with respective fluorophores: goat anti-mouse Alexa fluor 555 (Beyotime, China) and goat anti-rabbit Alexa fluor 488 (Beyotime, China). Fluorescence was assessed using an SP8 Leica Confocal Microscope.

### Nude xenograft mice model

Thirty BALB/c female nude mice, aged between 4 and 6 weeks, were purchased from the Institute of Experimental Animals of Guangxi Medical University and randomly divided into the NC agomir and miR-30b-5p agomir groups. B-CPAP cells were injected into the right axilla of mice at a concentration of 5 × 10^7^cells/mL (200 µ L/mouse). After 3 weeks, the intratumoral injection of 100 nM miRNA agomir was administered in the mice every 4 days for 2 weeks. Every 4 days, tumour xenograft growth was measured with calipers, and volume was calculated according to the following formula: tumor volume (Mm^3^) = (length × width)^2^ × 0.5. After 40 days, the nude mice were euthanized by overdose sodium pentobarbital at 300 mg/kg, and the tumor tissues were taken for immunohistochemistry and western blot assays. These animal experiments were approved by the Ethical Committee for Animal Utilization and Protection of Guangxi Medical University (202007052, 2020).

### Statistical analyses

All data (three biological repeats or samples) were expressed as the mean ± standard deviation (SD). SPSS 20.0 software (IBM, Chicago, IL, USA) and GraphPad Prism 7.0 (GraphPad Software Inc., CA, USA) was used for statistical analysis. T-test and one-way ANOVA were used for comparison between the groups. P < 0.0001 is indicated as ****P < 0.001 as ***P < 0.01 as **, and P < 0.05 as *. All the experiments were repeated thrice.

## Results

### MiR-30b-5p is down-regulated in PTC and is related to poor clinical features

**Fig. 1:**
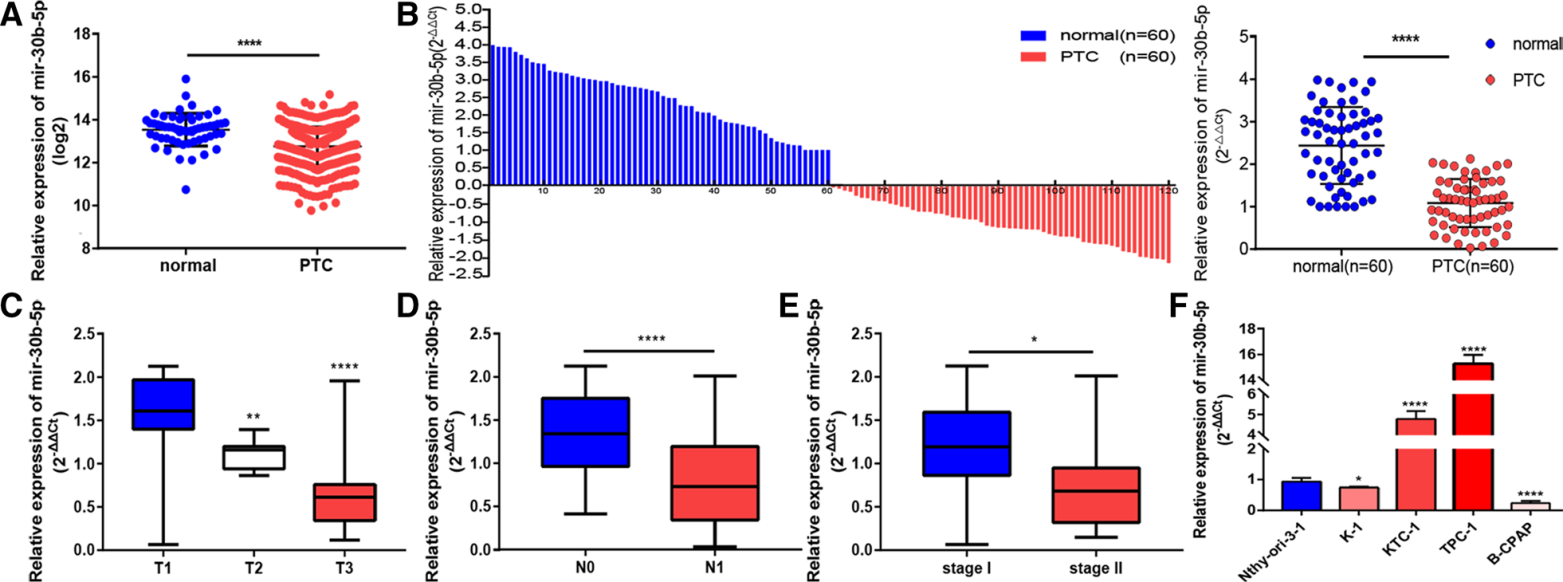
Mir-30b-5p is significantly down-regulated in thyroid carcinoma and is related to the progression of thyroid cancer. **A** Analysis of mir-30b-5p expression levels in papillary thyroid carcinoma using TCGA data. **B** Quantitative RT-PCR was used to detect the relative expression of mir-30b-5p in 60 paired papillary thyroid carcinoma tissues and paired normal tissues. **C** The relative expression level of mir-30b-5p in T1 stage, T2 stage and T3 stage of papillary thyroid carcinoma patients. **D** There was a significant difference in the expression of mir-30b-5p in papillary thyroid carcinoma patients with or without lymph node metastasis. **E** The relative expression level of mir-30b-5p in papillary thyroid carcinoma patient with stage I and stage II. **F** The relative expression of mir-30b-5p in 4 thyroid cancer cell lines and immortalized normal thyroid epithelial cell line Nthyr-ori-3-1 was analyzed by qRT-PCR. Data are presented as the mean ± SD (*P < 0.05, **P < 0.01, ***P < 0.001, ****P < 0.0001)

The analysis of the THCA dataset from the TCGA database shows that the expression of mir-30b-5p is low in PTC (Fig. 1A). To further verify this result, the qRT-PCR assay was used to evaluate the expression of mir-30b-5p in 60 pairs of PTC and paracancerous tissues. The results showed that the expression level of mir-30b-5p in PTC was lower than that of the paracancerous tissues (Fig. 1B; Additional file 1). 60 tumor tissues were divided into high expression and low expression groups to explore the correlation between the expression level of mir-30b-5p and clinicopathological features (Additional file 2: Table S1). It was found that the expression of mir-30b-5p was significantly correlated with the T stage (T1vs.T2, P = 0.0047; T1vs.T3, P < 0.0001), lymph node metastasis (N0 vs. N1, P < 0.0001), and tumor stage (I vs. II, P = 0.0214) (Fig. 1C–E). Also, we detected the expression of mir-30b-5p in normal thyroid cells (Nthy-ori-3-1) and four PTC cells (TPC-1, KTC-1, K-1, and B-CPAP). The results showed that compared to the normal thyroid cells, the expression level of mir-30b-5p in K-1 and B-CPAP cells was down-regulated (Fig. 1F, P  = 0.0232 and P < 0.0001; Additional file 1) while the expression level of mir-30b-5p in TPC-1 and KTC-1 cells was up-regulated (Fig. 1F, P < 0.0001 and P < 0.0001; Additional file 1). Therefore, K-1 and B-CPAP cell lines were used for all the subsequent studies, and the results suggested that the down-regulation of mir-30b-5p may be related to the development of PTC.

### Mir-30b-5p inhibits proliferation and causes apoptosis of PTC cells in vitro

**Fig. 2:**
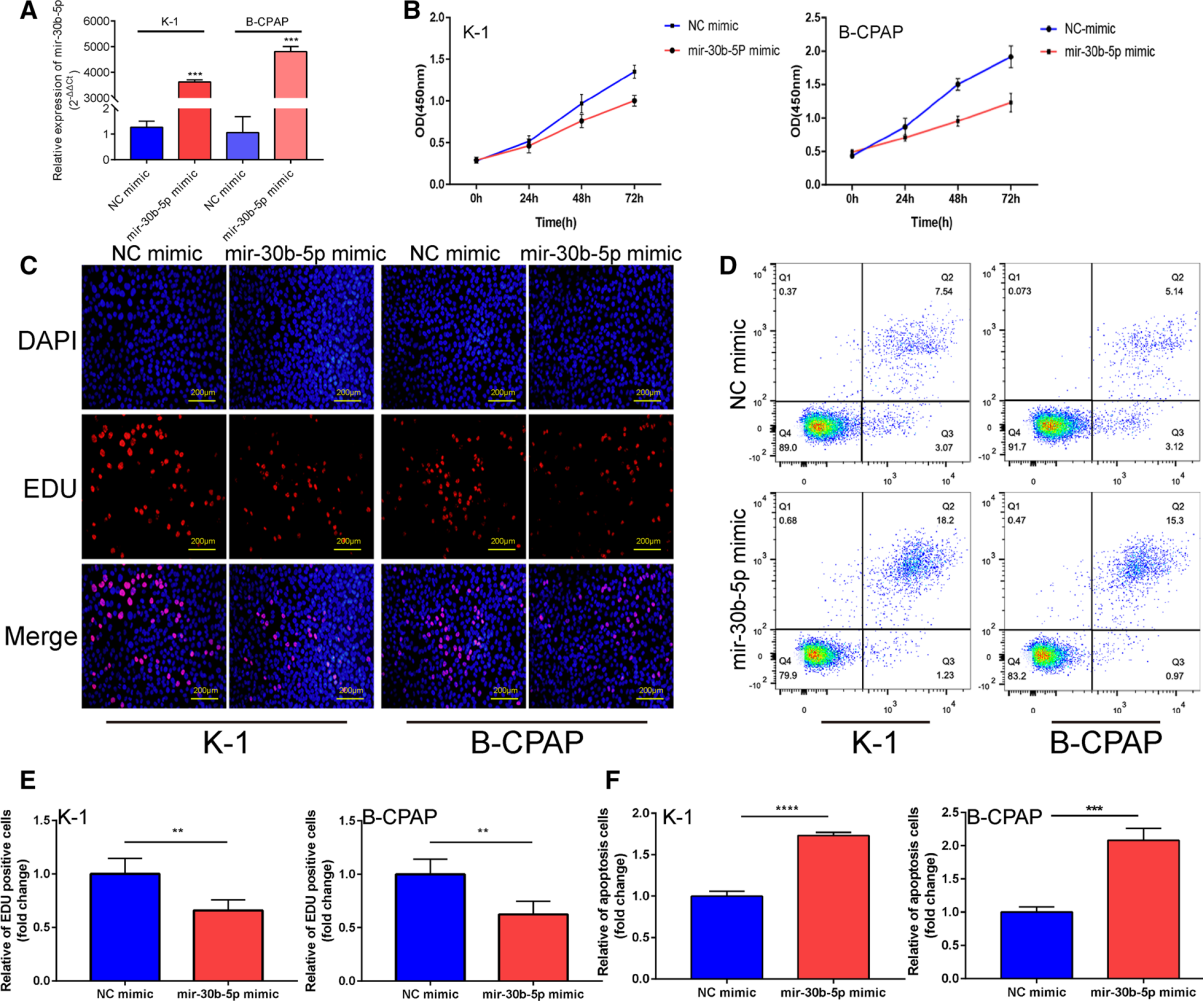
Overexpression of mir-30b-5p inhibited PTC cell proliferation and promotes apoptosis. **A** The relative expression level of mir-30b-5p was significantly up-regulated by mir-30b-5p mimic. **B** Cell proliferation was detected in both PTC cells after transfection of mir-30b-5p mimic by CCK8 kit. **C** Representative images of EdU assay. **D** Q1, Q2, Q3 and Q4 zones represent necrosis, late apoptosis, early apoptosis and healthy cells, respectively. **E** The relative fold changes of EdU positive cells were detected mir-30b-5p mimic. **F** The relative fold changes of apoptosis-positive cells were detected mir-30b-5p mimic. Data are presented as the mean ± SD (*P < 0.05, **P < 0.01, ***P < 0.001, ****P < 0.0001)

Mir-30b-5p mimic was transfected into K-1 and B-CPAP cells, respectively. Significant overexpression of miR-30b-5p in K-1 and B-CPAP cells was verified using qRT-PCR (Fig. 2A,  P =  0.0002 and P = 0.0006; Additional file 1). We first investigated the effects of overexpression of mir-30b-5p on PTC cell proliferation. Following the miR-30b-5p overexpression, the proliferative capability of PTC cell lines was investigated using the CCK8 assay (Fig. 2B; Additional file 3) and EdU staining (Fig. 2C, E, P = 0.0026, and P = 0.0020; Additional file 4), which showed a decrease in the proliferation. Moreover, the number of apoptotic cells in the miR-30b-5p mimic group was found to be significantly more than that of the NC mimic group (Fig. 2D, F,  P < 0.0001 and P = 0.0007). These results suggested that the miR-30b-5p regulated the proliferation and apoptosis of PTC cells.

### Mir-30b-5p suppresses migration and invasion of the PTC cells in vitro

**Fig. 3:**
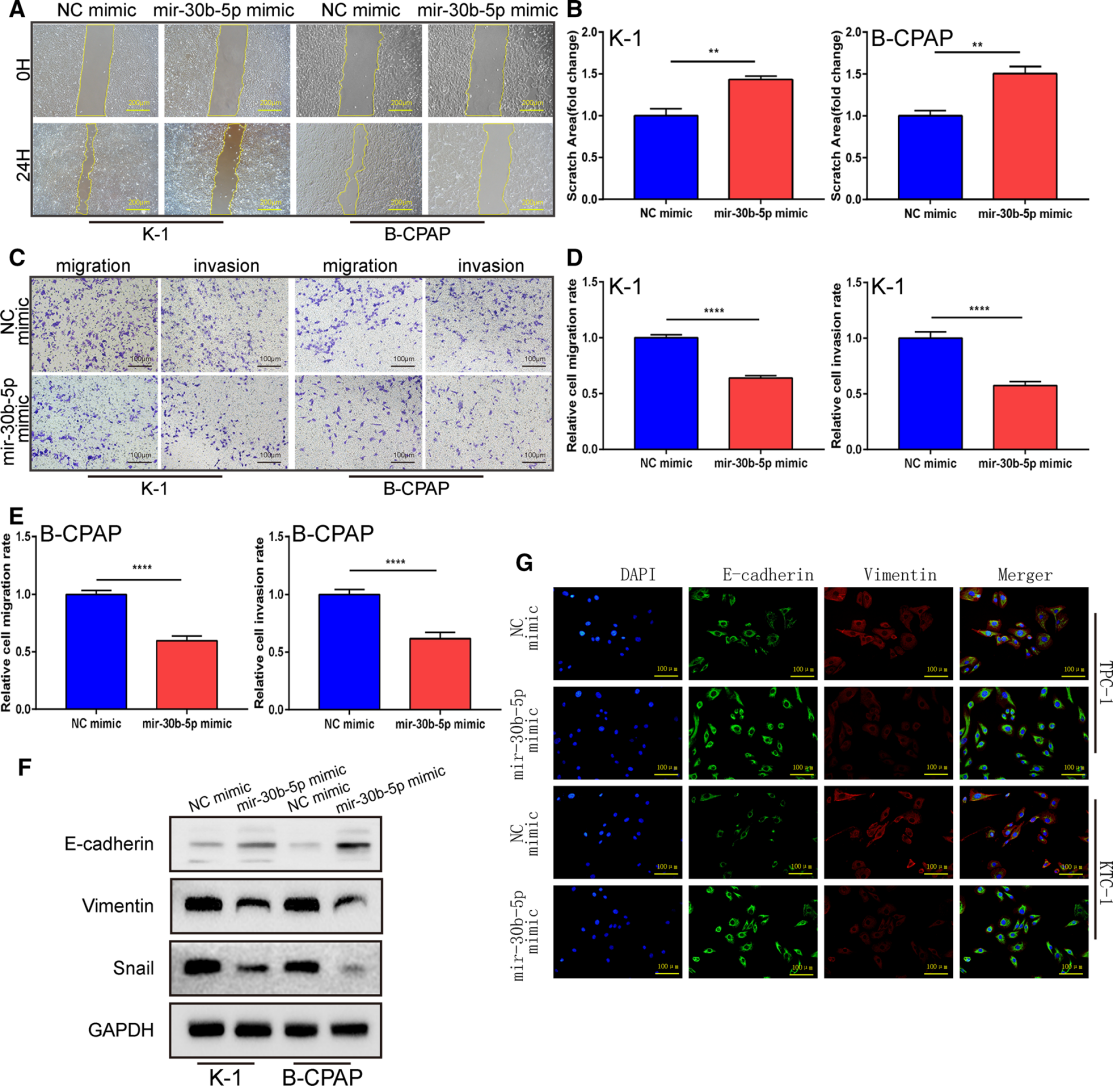
Overexpression of mir-30b-5p inhibited PTC cell migration, invasion and induces the MET. **A** Scratch assay following mir-30b-5p mimic transfected. **B** Quantification of A. **C** Transwell migration and matrigel invasion assays. **D**, **E** Quantification of transwell assay. **F** Western blotting assay showing the protein expression levels of EMT markers in the indicated groups. **G** Immunofluorescence staining against EMT markers E-cadherin and Vimentin. Data are presented as the mean ± SD (*P < 0.05, **P < 0.01, ***P < 0.001, ****P < 0.0001)

To further analyze the biological behavior of mir-30b-5p in PTC, the migratory and invasive ability of PTC cells were measured using the wound healing and transwell assays. Wound healing assays showed that the overexpression of miR-30b-5p notably inhibited the PTC cell migration (Fig. 3A, B; P = 0.0013 and P = 0.0011). Transwell migration and invasion assays also indicated that miR-30b-5p markedly decreased the number of migrated and invaded cells in K-1 and B-CPAP cell lines (Fig. 3C–E, P < 0.0001; Additional file 5). It is generally believed that EMT is the key process in tumor migration, invasion, metastasis, and spread. Thus, we tested whether miR-30b-5p is involved in EMT (Epithelial-mesenchymal transition). For the protein expression of EMT markers, western blotting was used to verify the representative EMT markers (E-cadherin, snail, and vimentin). The results showed that the overexpression of mir-30b-5p decreased the expression of mesenchymal markers, including snail and vimentin while increasing the expression of epithelial marker E-cadherin (Fig. 3F; Additional file 6). The changes in the EMT markers, namely, E-cadherin and Vimentin, were further confirmed by the IF analysis. These data suggested that the overexpression of miR-30b-5p can effectively inhibit the metastatic characteristics of PTC cells.

### MiR-30b-5p directly targets GALNT7

**Fig. 4:**
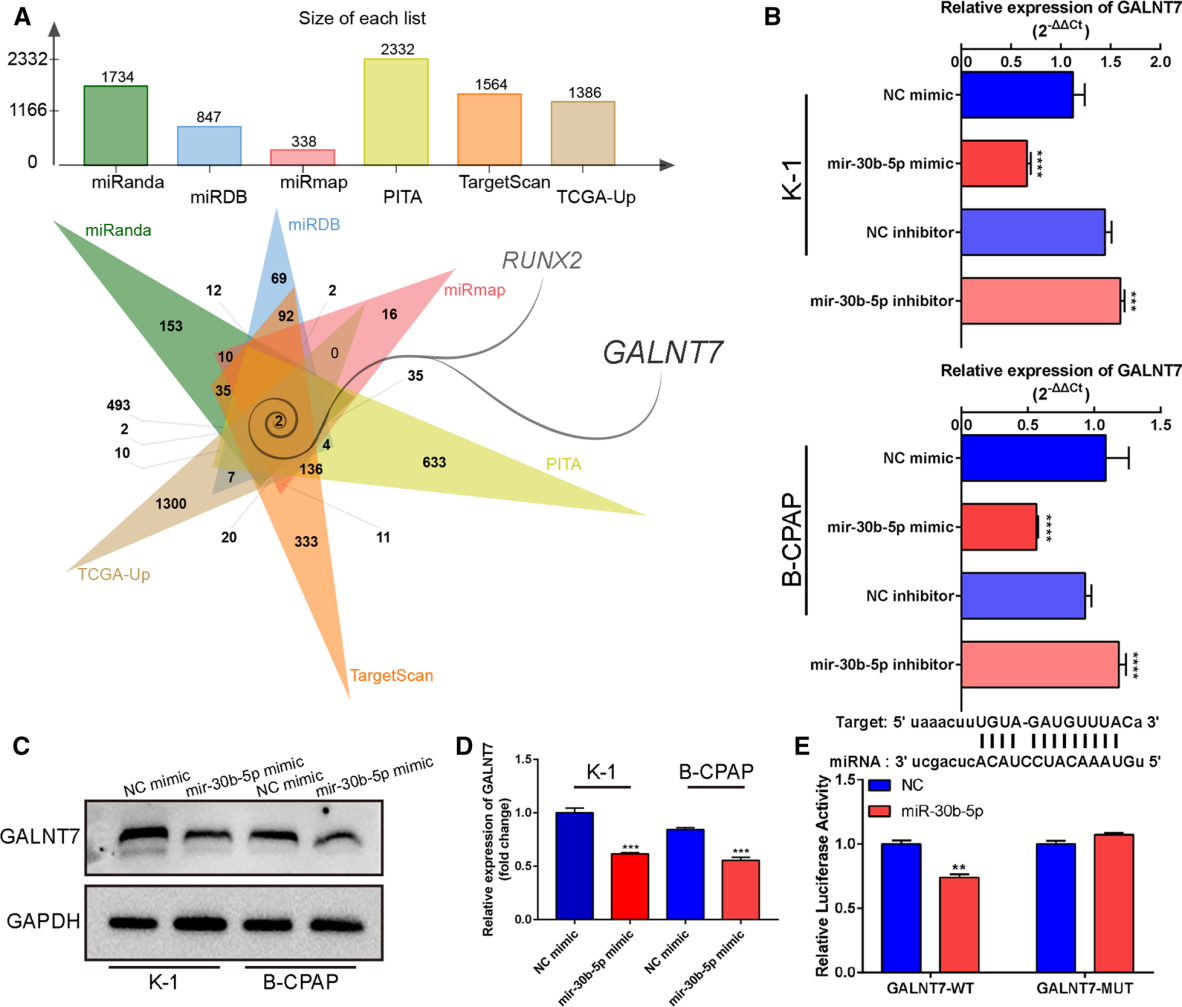
GALNT7 is a direct target of mir-30b-5p. **A** Identification of 2 commonly changed targeted mRNAs of mir-30b-5p from the five publicly profile datasets (TargetScan, miRDB, miRmap, PITA and miRanda) and TCGA datasets. Different color areas represented different datasets. The cross areas meant the commonly changed mRNAs of mir-30b-5p. The height of the column represents the predicted number of genes. **B** Validation of GALNT7 target genes by real-time PCR. **C** Western Blot confirms downregulation of GALNT7 protein levels in the mir-30b-5p mimic group. **D** Quantification of **C**. **E** Mir-30b-5p mimic prominently reduced luciferase activity in GALNT7-wild, not in GALNT7-mut. Data are presented as the mean ± SD (*P < 0.05, **P < 0.01, ***P < 0.001, ****P < 0.0001)

To clarify the potential mechanism underlying the miR-30b-5p function, we used TargetScan, miRDB, miRmap, PITA, and miRanda websites to explore the downstream targets of miR-30b-5p. The gene sets were selected based on their overlapping with the upregulated genes in the TCGA-THCA tumor samples for subsequent studies, with the Venn diagram showing a total of two genes being included (Fig. 4A). Here, we chose the target gene GALNT7 for a follow-up study. Subsequently, PTC cell lines were transfected with both the miR-30b-5p mimic and miR-30b-5p inhibitor. After 24 h, the qRT-PCR assay was used to detect the expression levels of GALNT7. The results showed that the expression of GALNT7 was found to be lower in the mir-30b-5p mimic group compared to that of the NC mimic group, while we obtained opposite results in the miR-30b-5p inhibitor and NC inhibitor groups (Fig. 4B, P < 0.0001, P = 0.0007, P < 0.0001 and P < 0.0001; Additional file 1). This result was also confirmed by western blot assay, which showed that the level of GALNT7 protein decreased significantly after the overexpression of mir-30b-5p in K-1 and B-CPAP cell lines (Fig. 4C, D; P = 0.0001 and P = 0.0001; Additional file 6). These results suggested that GALNT7 may be a potential target for miR-30b-5p. To confirm that GALNT7 is the direct target of miR-30b-5p, we designed a luciferase reporter gene with wild type (Wt) or mutant (Mut) 3'-UTR in the GALNT7 gene. Luciferase reporter gene analysis showed that the relative luciferase activity in the miR-30b-5p and GALNT7-WT cotransfected cells was significantly lower than that of the control group (Fig. 4E, P  = 0.0032; The detailed site mutation information is in Additional file 7). This luciferase activity is canceled once a mutation occurs at a potential miR-30b-5p binding site. These results showed that the GALNT7 is the direct target of miR-30b-5p, and the expression of GALNT7 in PTC cells is negatively regulated by miR-30b-5p.

### GALNT7 overexpression rescues the aggressive characteristics of PTC cells inhibited by miR-30b-5p

**Fig. 5:**
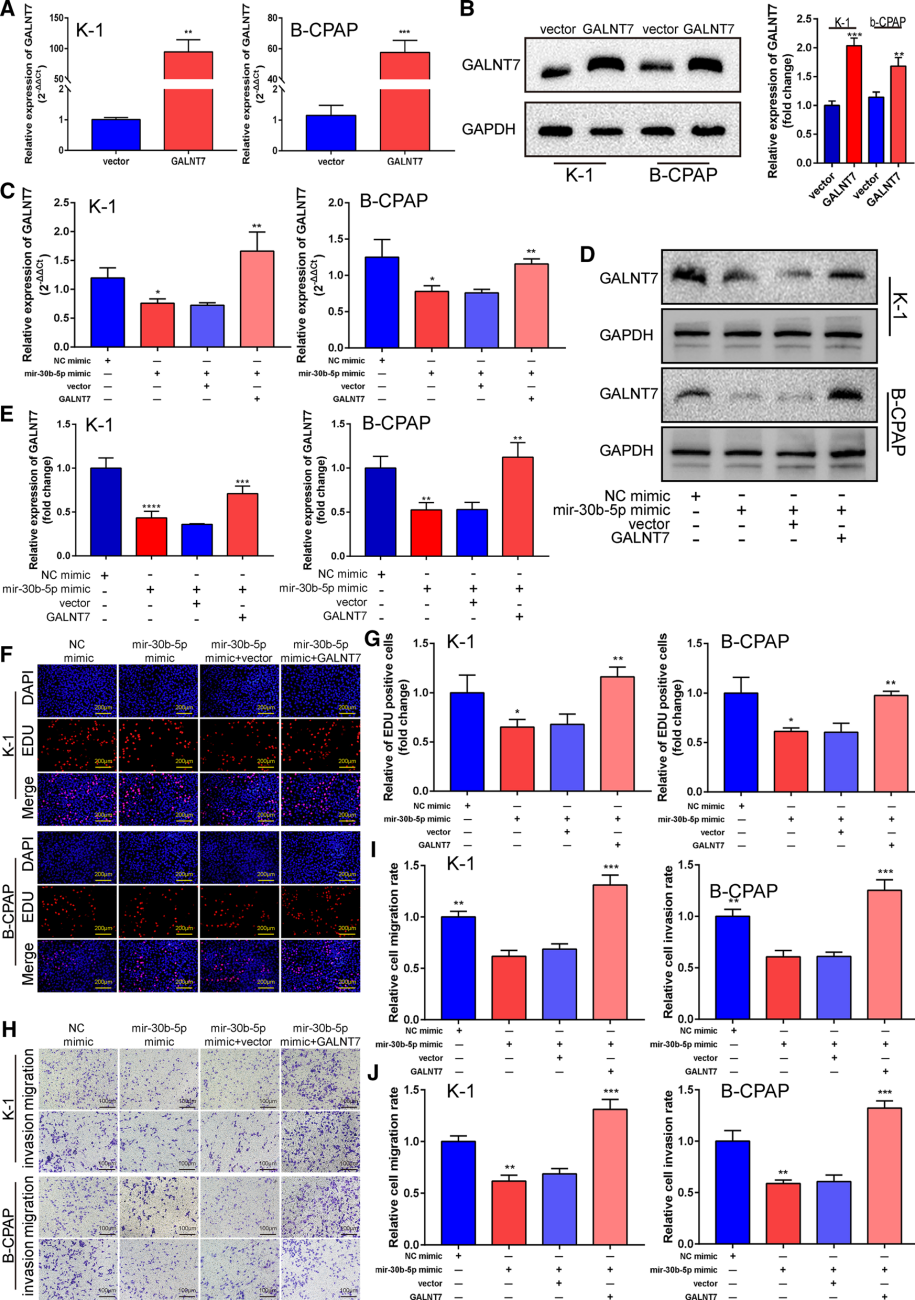
Mir-30b-5p regulates proliferation and metastasis by targeting GALNT7. **A** Quantitative RT-PCR was used to detect the relative expression of GALNT7 after cells transfected with empty vector or pcDNA-GALNT7. **B** The protein levels of GALNT7 were detected after transfection with empty vector or pcDNA-GALNT7. Right panel, quantification of the result. **C**–**E** K-1 and B-CPAP cells were transfected with NC-mimic, miR-30b-5p mimic, or cotransfected with miR-30b-5p mimic and Vector, miR-30b-5p mimic and pcDNA-GALNT7.The mRNA and protein levels of GALNT7 were detected using qRT-PCR and western blot, respectively. **F** EdU was used to detect cell proliferation after transfection of NC-mimic, miR-30b-5p mimic or co-transfection of miR-125a-5p mimic and Vector, miR-125a-5p mimic and pcDNA-GALNT7. **G** Quantification of F. **H** Cell migration and invasion were detected using the Transwell assay after transfection with NC-mimic, miR-30b-5p mimic or co-transfection with miR-125a-5p mimic and Vector, miR-125a-5p mimic and pcDNA-GALNT7. **I**, and **J** Quantification of **H**. Data are presented as the mean ± SD (*P < 0.05, **P < 0.01, ***P < 0.001, ****P < 0.0001)

To verify that GALNT7 is the functional target of miR-30b-5p, we carried out rescue experiments in the PTC cells. The overexpression plasmid was transferred into the PTC cell lines, where the negative control group was the vector group along with the GALNT7 overexpression group and the GALNT7 group. The qRT-PCR and western blot assays were used to verify the transfection efficiency, and the results are shown in Fig. 5A (P = 0.0012 and P = 0.0002; Additional file 1) and B (P = 0.0003 and P = 0.0058; Additional file 6). When miR-30–5p mimic was transfected into K-1 and B-CPAP cells, a decrease was observed in the mRNA and protein levels of GALNT7 while the co-transfection of pcDNA-GALNT7 and miR-30b-5p mimics restored the mRNA and protein levels of GALNT7 (Fig. 5C, P = 0.0167, P = 0.0084, P = 0.0327, and P = 0.0013; Additional file 1) (Fig. 5D, E, P  = 0.0021, P = 0.0022, P = 0.0064, and P = 0.005; Additional file 6). Although miR-30b-5p inhibits the proliferation, invasion, and migration of PTC cells, GALNT7 recurrence can rescue the changes in the process. EdU staining showed that the transfection of pcDNA-GALNT7 could antagonize the decrease of cell proliferation induced by miR-30b-5p mimics (Fig. 5F, G, P = 0.0369, P = 0.0042, P = 0.0154 and P = 0.0031 Additional file 4). Transwell invasion and migration assay results suggested that the transfection of miR-30b-5p mimic weakens the metastatic ability of PTC cells while the co-transfection of pcDNA-GALNT7 and miR-30b-5p mimic can reverse this effect (Fig. 5H, I, P = 0.0011, P = 0.0006, P = 0.0018, and P = 0.0005; Additional file 5). The results of quantifications are shown in Fig. 5J (P = 0.0016, P = 0.0004, P = 0.0028, and P = 0.0002). Overall, these results indicated that GALNT7 is a functional target of miR-30b-5p, which inhibits the malignant behavior of PTC cells by down-regulating GALNT7.

### Knockdown of GALNT7 suppresses the aggressive characteristics of PTC cells

**Fig. 6:**
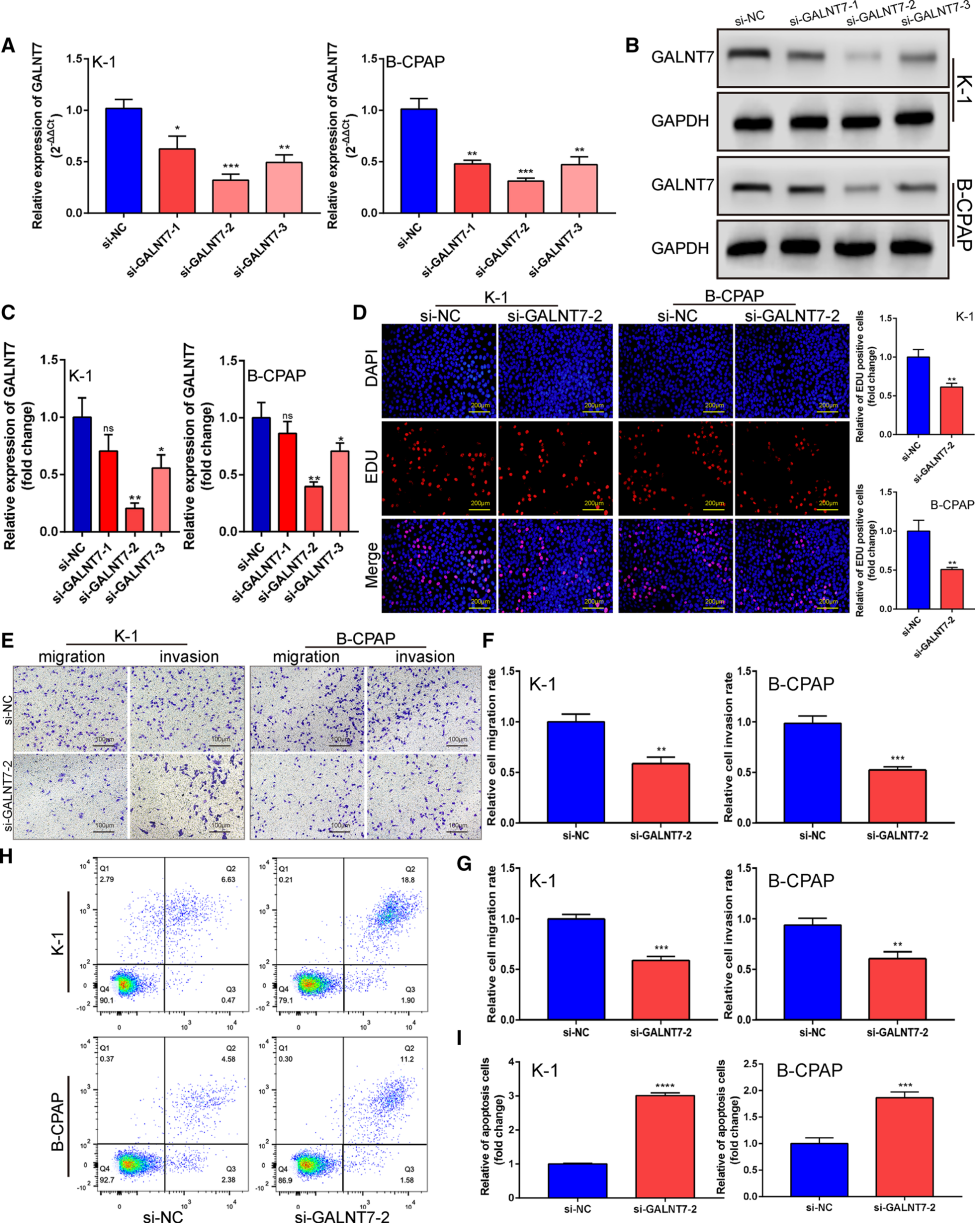
Down-regulation of GALNT7 inhibited PTC cell proliferation and promotes apoptosis. **A**, **B** qRT-PCR and western blot assays were used to verify the silencing effect of siRNA. **C** Quantification of **B**. **D** The effect of silencing GALNT7 on cell proliferation was detected by EdU. Right panel, quantification of the result. **E** Transwell migration and matrigel invasion assays. **F**, **G** Quantification of **E**. **H** Apoptosis assay. **I** The relative fold changes of apoptosis-positive cells were detected. Data are presented as the mean ± SD (*P < 0.05, **P < 0.01, ***P < 0.001, ****P < 0.0001)

GALNT7 is a member of the glycosyltransferase family, the disorder of which has been found in many diseases [33, 36, 41–44]. The results from the TCGA database suggest that GALNT7 is highly expressed in PTC (Additional file 12). We used the siRNA to silence the expression of GALNT7 and observed whether knocking down GALNT7 showed the same function as miR-30b-5p. As shown in Fig. 6A–C, the mRNA and protein levels of GALNT7 decreased significantly after the transfection of si-GALNT7 (Additional files 1 and 6). This si-GALNT7-2 was then selected for subsequent experiments. EdU staining showed that the proliferation of K-1 and B-CPAP cells was significantly inhibited after silencing GALNT7 (Fig. 6D, P = 0.0035 and P = 0.0037; Additional file 4). The invasion and migration experiments showed that the number of migration and invasion of the cells decreased significantly in GALNT7 knocked-out PTC cells (Fig. 6E–G, P  = 0.0020, P = 0.0005, P = 0.0003, and P = 0.0035; Additional file 5). Also, the downregulation of GALNT7 caused more apoptosis of tumor cells (Fig. 6H, I, P < 0.0001 and P = 0.0006). These results suggested that the knockout of the GALNT7 gene showed consistency with the overexpression of miR-30b-5p.

### GALNT7 promotes cell proliferation and invasion by activating EGFR/PI3K/AKT pathway

**Fig. 7:**
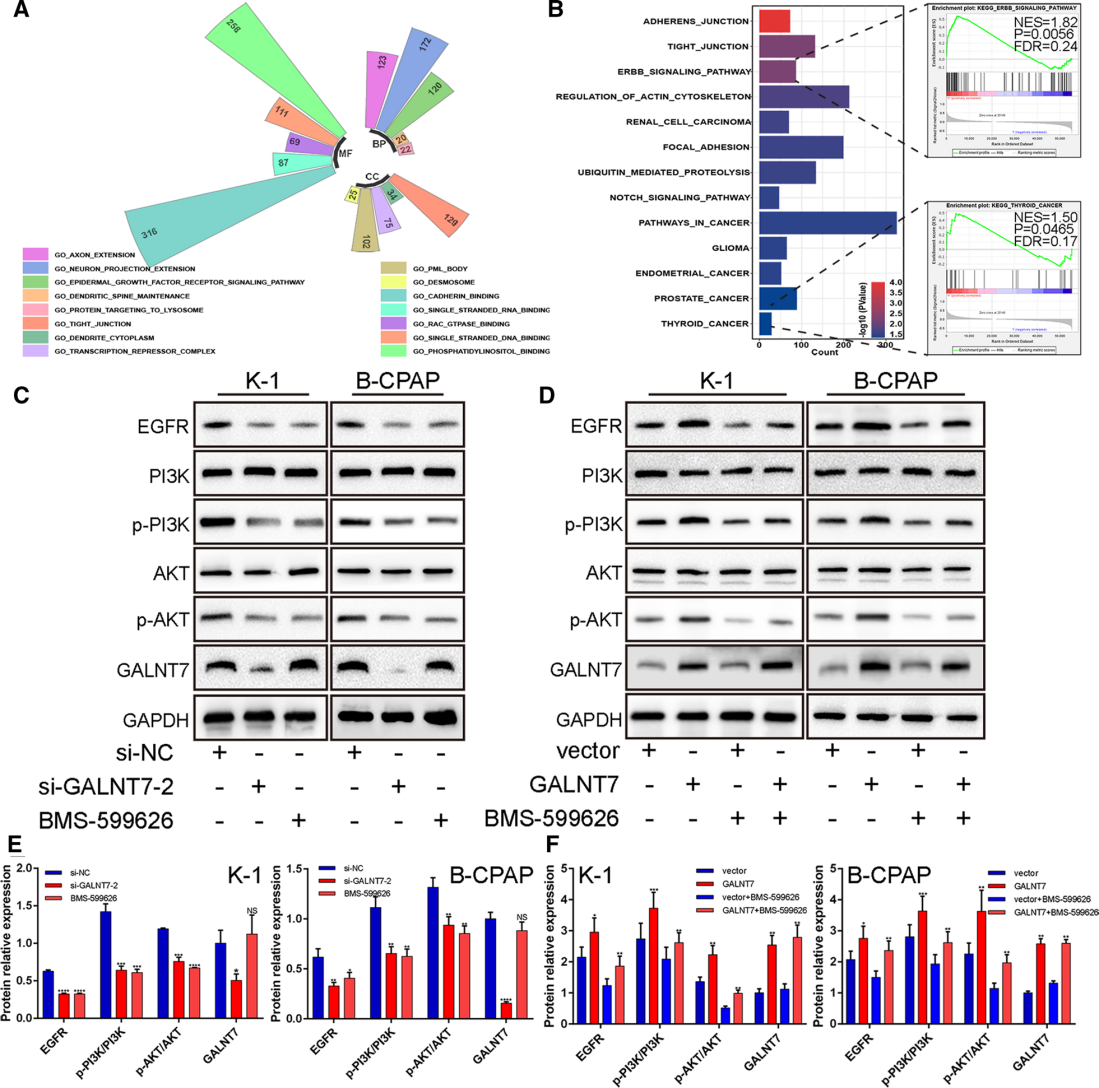
GALNT7 promotes cell proliferation and invasion by activating EGFR/PI3K/AKT pathway. **A**, **B** GO and KEGG analysis was performed using the GSEA software. **C**, **D** Western blot analysis was used to detect the protein expression levels of EGFR, PI3K, p-PI3K, AKT, p-AKT and GALNT7 after transfection respectively. **E** Quantification of **C**. **F** Quantification of **D**. Data are presented as the mean ± SD (*P < 0.05, **P < 0.01, ***P < 0.001, ****P < 0.0001)

**Fig. 8:**
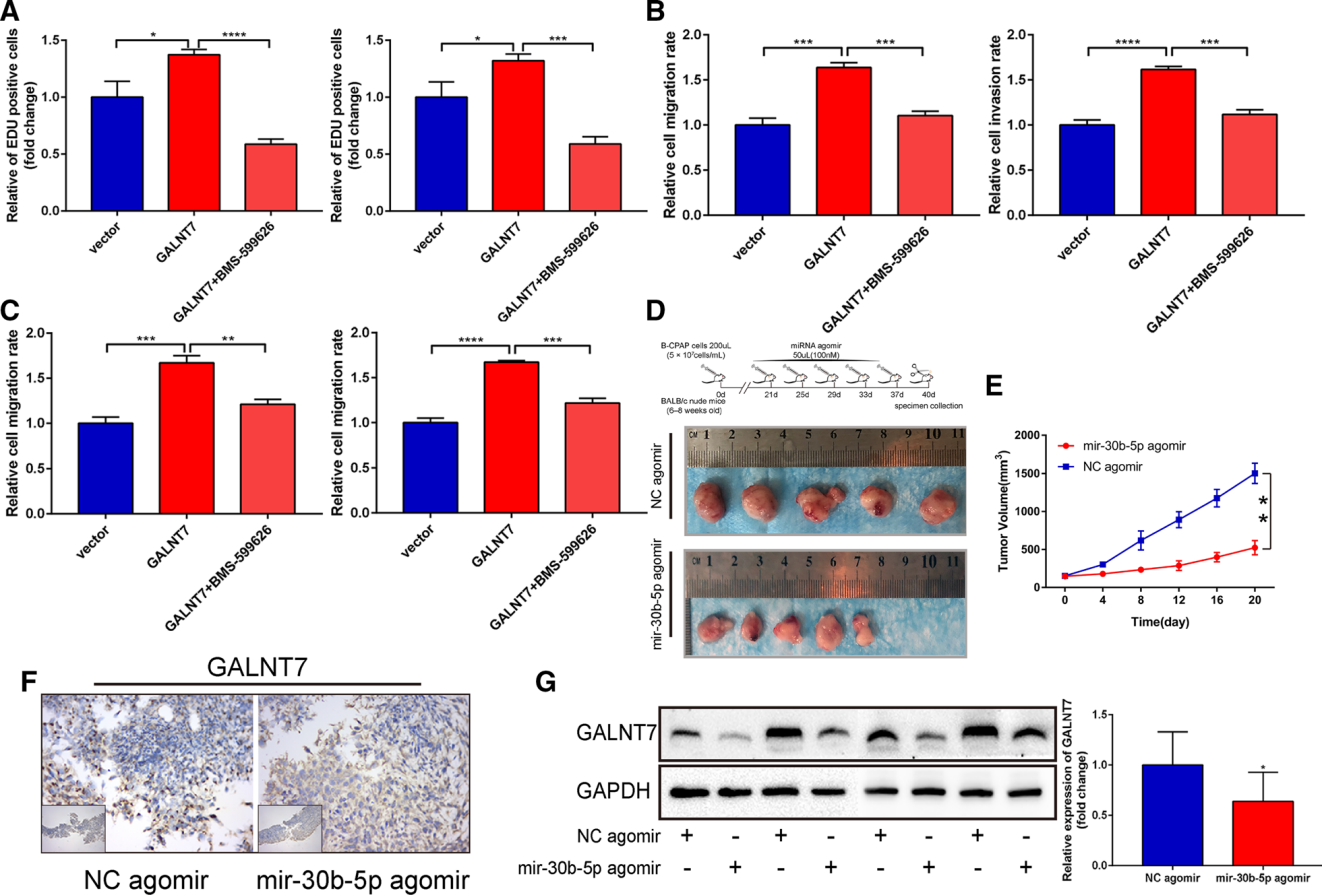
The repression of the EGFR/PI3K/AKT pathway impair the ability of proliferation and metastasis in PTC cell. Overexpression of mir-30b-5p inhibits tumor progression in vivo. **A** The relative fold changes of EdU positive cells were detected with pcDNA-GALNT7 or BMS-599626. **B**, **C** The relative fold changes of invasion and migration cells were detected pcDNA-GALNT7 or BMS-599626. **D**, **E** Overexpression of mir-30b-5p inhibited the growth of transplanted B-CPAP cell in nude mice. **F**, **G** Immunohistochemistry and Western Blot were used to detect the expression after miRNA agomir was injected into mice. Data are presented as the mean ± SD (*P < 0.05, **P < 0.01, ***P < 0.001, ****P < 0.0001)

Using the TCGA transcriptome data, we performed the GSEA analysis on the signaling pathways that GALNT7 might have been involved in. The results suggested that the EGFR-related signaling pathways were significantly enriched (Fig. 7A and B; Detailed data in Additional file 9). Therefore, we detected the activity of the EGFR/PI3K/AKT pathway in K-1 and B-CPAP cells and used selective HER1 and HER2 inhibitors (BMS-599626, 10 µM) to inhibit the activity of the pathway. As shown in Fig. 7C and E, the levels of major signaling molecules were significantly lower in the EGFR/PI3K/AKT pathway in the PTC cells treated with BMS-599626 or si-GALNT7–2 than those found in the negative control group (Additional file 6). Similarly, the expression levels of EGFR, p-PI3K, and p-AKT were higher in PTC cells transfected with pcDNA-GALNT7 than those observed in the control group. After the pathway was blocked by BMS-599626, the expression of EGFR, p-PI3K, p-AKT in the GALNT7 group did not show any further increase. These results are shown in Figs. 7D and F (Additional file 6). Accordingly, the inhibition of the EGFR/PI3K/AKT signaling pathway reduced the proliferation and invasion ability of the PTC cells (Fig. 8A–C; Additional files 4 and 5). Overall, these data suggested that the GALNT7 plays an important role in the progression of PTC through the EGFR/PI3K/AKT pathway.

### Overexpression of miR-30b-5p suppressed the tumor growth of PTC and the expression of GALNT7 in vivo

To study whether miR-30b-5p has an anti-tumor effect in vivo, we established a xenograft tumor model. The results showed that the tumor volume of the miR-30b-5p agomir group was significantly smaller than that of the control group and NC agomir group (Fig. 8D and E; P = 0.0022; Additional file 8). Additionally, compared to the NC agomir group, the protein expression of GALNT7 was found to be decreased in the mir-30b-5p agomir group (Fig. 8F and G; Additional file 6). These results suggested that miR-30b-5p may inhibit the growth of PTC in vivo by inhibiting the expression of GALNT7.

## Discussion

MicroRNAs (miRNAs) are an important regulator of gene expression, and their disorder plays a vital role in cancer. Although few reports describe miRNA with carcinogenic function, it is generally agreed that miRNA deletion promotes tumorigenesis [45]. We included 60 pairs of PTC tissues and paired tissue samples in the clinical test to explore the expression pattern of miR-30b-5p and found that the expression of miR-30b-5p was down-regulated in the PTC. The low expression of miR-30b-5p in PTC patients was positively correlated with the progression of the TNM stage. Interestingly, we found that miR-30b-5p was not always lowly expressed in thyroid cancer cell lines. The expression of miR-30b-5p in TPC-1 and KTC-1 was significantly higher than that in normal thyroid cell lines, K-1 and B-CPAP cell lines. Since both K-1 and B-CPAP are braf mutant cell lines, we speculate that the abnormal result may be related to braf mutation. Subsequently, the results of TCGA-THCA data analysis supported our conjecture, which suggested that the expression of miR-30b-5p was lower in the population with braf mutations (Additional file 11; the difference was not statistically significant). These results suggest that the expression level of miR-30b-5p may be affected by braf mutation. It will be significant to verify the relationship between braf mutation and miR-30b-5p expression level in the future.

An increasing number of studies have indicated that most miRNAs regulate their target genes by repressing their expression levels [46]. As a tumor suppressor, miR-30b-5p regulates the proliferation, metastasis, and EMT of renal cancer cells by down-regulating the expression of GNA13 [12]. In colon cancer, miR-30b-5p inhibits metastasis by targeting Rap1b [14]. MiR-30b-5p can inhibit the proliferation and cell cycle of hepatocellular carcinoma cell lines [47]. MiR-30b-5p inhibits the migration and invasion of esophageal cancer by targeting ITGA5 and PDGFRb [48]. In addition, the miR-30b-5p inhibits lung cancer progression and enhances cisplatin sensitivity by targeting LRP8 [13]. This study shows that miR-30b-5p can significantly inhibit the proliferation, migration, and invasion of PTC cells while promoting the apoptosis of PTC cells. It is similar to the results of previous studies. Our results also suggest that GALNT7 is the downstream target gene of miR-30b-5p, which (Polypeptide *N*-acetylgalactosaminyltransferase 7) is a member of the GalNAc-transferase family. The enzyme encoded by this gene controls the initiation step of mucin-type O-linked protein glycosylation, and it also controls the transfer of *N*-acetylgalactosamine to serine and threonine amino acid residues [49]. Abnormal expression of GALNT7 has been found in many types of cancer, and MiR-125a-5p is shown to inhibit the formation of cervical cancer by inhibiting the expression of GALNT7 in vivo [36]. The high expression of GALNT7 is related to the poor prognosis of gliomas and can be used as an effective biomarker in gliomas [43]. The expression of GALNT7 in laryngeal carcinoma cells is negatively regulated by miR-34a and miR-34c, thus, playing a tumor inhibitory role [32]. SNHG7/miR-34a may be involved in the progression of colorectal cancer through the GALNT7 pathway [42]. The expression of GALNT7 in cervical cancer is often upregulated and promotes tumor proliferation [44]. Most importantly, experimental studies do not consistently support the role of GALNT7 in the tumor. The ectopic expression of miR-30b/30d has been reported to promote the metastasis of melanoma cells by directly targeting GALNT7 along with increasing the synthesis of immunosuppressive cytokine IL-10 while reducing the activation and recruitment of immune cells [50]. We evaluated the effect of GALNT7 on tumor behavior in PTC, and according to our experimental results, GALNT7 can promote the proliferation and metastasis of papillary thyroid cancer cell lines while silencing the expression of GALNT7 can promote the apoptosis of PTC cells. We believed that miR-30b-5p functioned through GALNT7, and to make this prediction convincing, we examined the expression of GALNT7 after down-regulation or overexpression of miR-30b-5p. The results of the rescue experiment showed that the inhibitory effect of miR-30b-5p on PTC cells could be neutralized by GALNT7. Also, we designed a luciferase experiment to confirm the targeted binding effect between mir-30b-5p/GALNT7. Convincingly, the constructed luciferase experiment proved that GALNT7 was the target of miR-30b-5p. These results confirmed that miR-30b-5p affects the progression of PTC cells by directly regulating GALNT7.

Previous reports have suggested that GALNT7 is involved in the regulation of tumor progression through PI3K/Akt/mTOR [42] and EGFR/PI3K/AKT [36] pathways. To determine the potential molecular mechanism of GALNT7 activity in PTC cells, we used bioinformatics to analyze the signaling pathways that GALNT7 might be involved in. The results showed that the EGFR-related signaling pathways were enriched many times, which was similar to the results of the previous studies. Many reports believe that the high expression of EGFR is positively correlated with the progress of PTC [22, 24, 25, 51, 52]. And the activation of the EGFR signal pathway contributes to the development of EMT in tumor cells [27, 28, 53]. Our research showed that GALNT7 positively regulated the EGFR/PI3K/AKT pathway, and the selective inhibitor BMS-599626 could weaken the promoting effect of GALNT7 on the proliferation and invasion capacity of PTC cells. When the inhibitors blocked the EGFR signal, it decreased the proliferation and invasiveness of PTC cells. GALNTs initiate the sequence by adding *N*-acetylgalactosamine (GalNAc) to serine or threonine residues, resulting in a structure called TN polysaccharides. It has been reported that malignant tumors lead to a sharp increase in TN levels, and this phenotype is the same in most types of solid malignant tumors, with a frequency of 70–90% [49, 54]. At present, the specific substrate of GALNT7 is not clear. Based on GSEA analysis, GALNT7 is involved in the modification of multiple small molecular, including cadherin binding, single-stranded RNA binding, Rac GTPase binding, single-stranded DNA binding, phosphatidylinositol binding, and Rho GTPase binding. In addition, the results of biological process enrichment analysis suggest that GALNT7 contributes to the transition from the G1 phase to the S phase in the mitotic cell cycle. These results may be the reason why GALNT7 increases the expression level of EGFR. The analysis result from TCGA datasets also provides strong evidence. The online analysis of GEPIA showed a significant positive correlation between the expression of GALNT7 and EGFR in TCGA-THCA datasets (This part of the data was shown in the Additional files 9, 10). These results suggest that GALNT7 up-regulates the expression of EGFR through a series of signal pathways. Of course, the modified substrate of GALNT7 is still worthy of further study. Anyway, this study shows that GALNT7 promotes the proliferation and metastasis of PTC cells by up-regulating the expression of EGFR and further activating the EGFR/PI3K/AKT pathway. However, there are still many shortcomings in this experiment. As mentioned earlier, the relationship between braf mutation and the expression level of miR-30b-5p needs to be verified, the modified substrate of GALNT7 needs to be identified, and how GALNT7 affects the process of cell cycle needs to be confirmed in the future.

## Conclusions

This study confirmed for the first time that mir-30b-5p/GALNT7 axis inhibits the proliferation and metastasis of PTC cells by regulating the EGFR/PI3K/AKT pathway, thus, providing a new target and better understanding of the possible pathogenesis. This result may help in designing molecular treatment strategies for PTC.

## Supplementary Information

**Additional file 1.** The raw data of qRT-PCR assays.

**Additional file 2.** The clinical characteristics of 60 patients with papillary thyroid carcinoma.

**Additional file 3.** The raw data of CCK8 assay.

**Additional file 4.** The quantitative data of EdU assay.

**Additional file 5.** The quantitative data of Transwell assay.

**Additional file 6.** The original result of western blot is provided as a PDF file.

**Additional file 7.** The raw data of Dual-Luciferase Reporter Assay.

**Additional file 8.** The tumor volume data in xenograft tumor model.

**Additional file 9.** The GSEA analysis on the signaling pathways.

**Additional file 10.** The results of correlation analysis of GALNT7 and EGFR.

**Additional file 11.** Differential expression of miR-30b-5p in patients with braf mutant and wild type thyroid carcinoma.

**Additional file 12.** The expression of GALNT7 in TCGA-THCA datasets.

## Data Availability

All data discussed are contained within the manuscript or the supporting material.

## Abbreviations

PTC: Papillary thyroid carcinoma
TCGA: The Cancer Genome Atlas
mRNA: Messenger RNA
miRNA: MicroRNA
DTCs: Differentiated thyroid carcinoma
GSEA: Gene set enrichment analysis
