# Supplementary material for: miR-30b-5p inhibits proliferation, invasion, and migration of papillary thyroid cancer by targeting GALNT7 via the EGFR/PI3K/AKT pathway
Source: Cancer Cell Int. 2021 Nov 24;21:618. doi: 10.1186/s12935-021-02323-x (PMC8611849; doi:10.1186/s12935-021-02323-x)

Fig.3 (F)

E-cadhrein

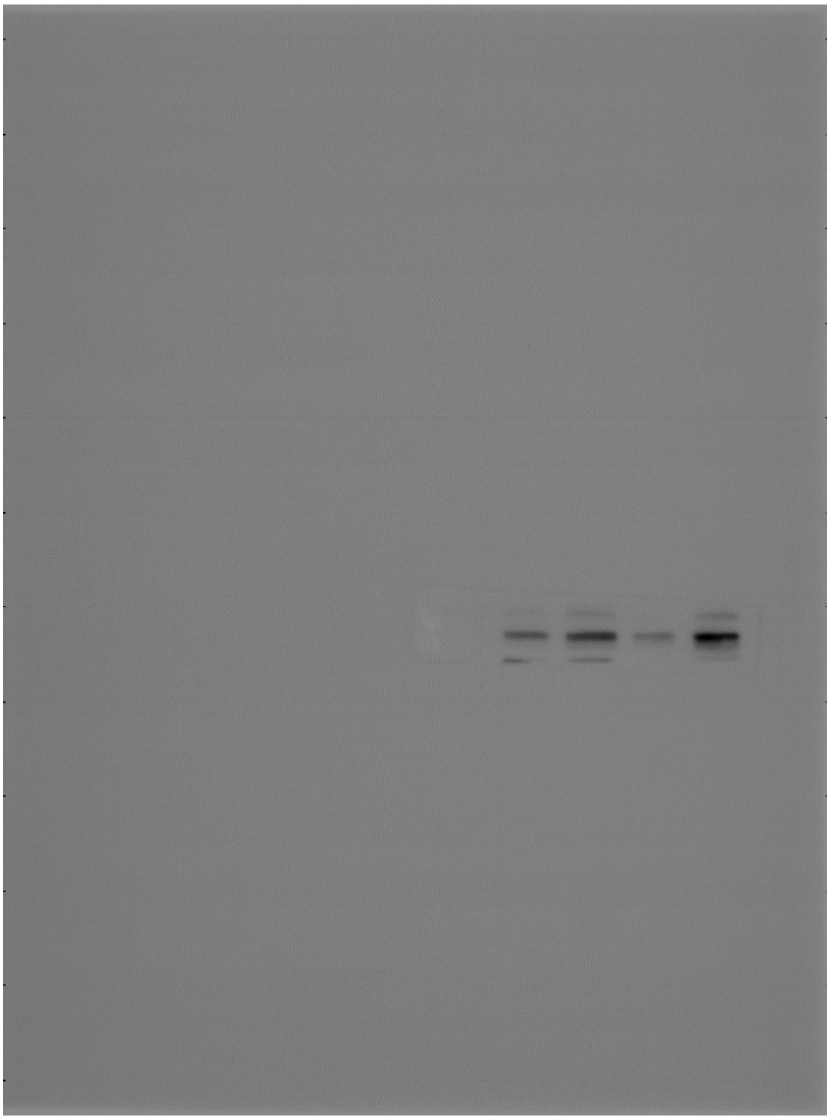

Vimentin

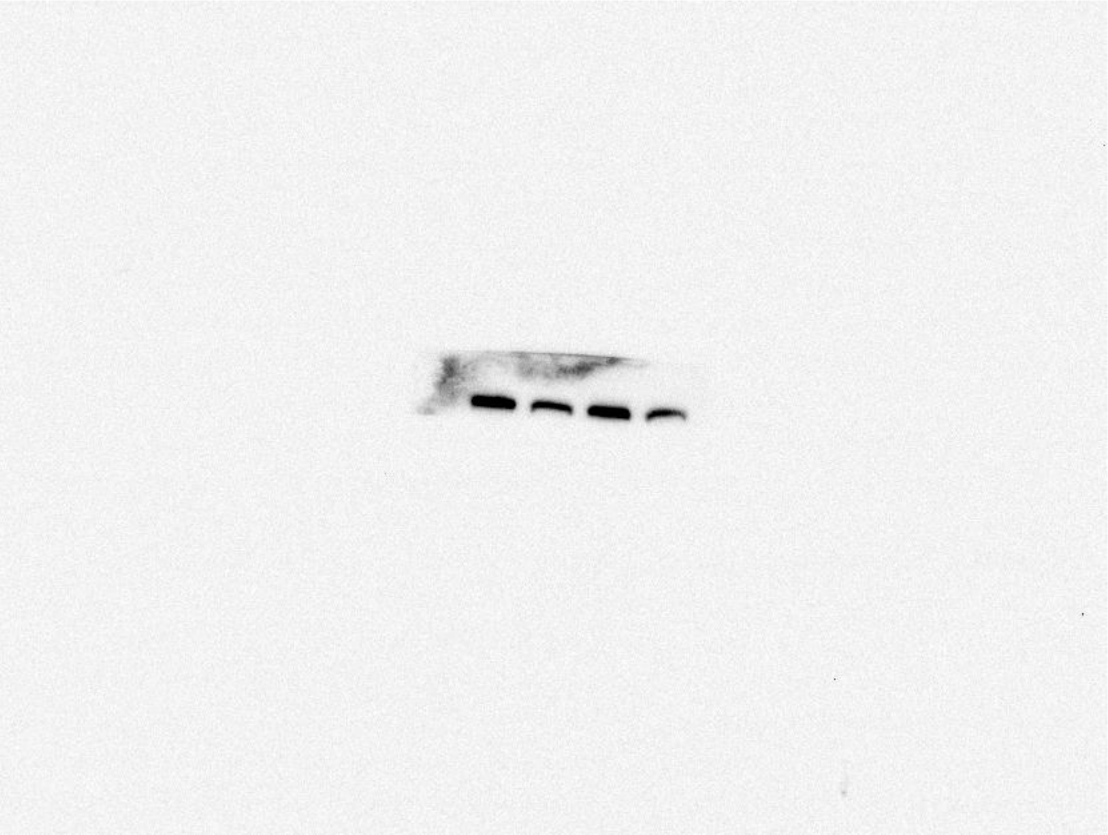

Snail

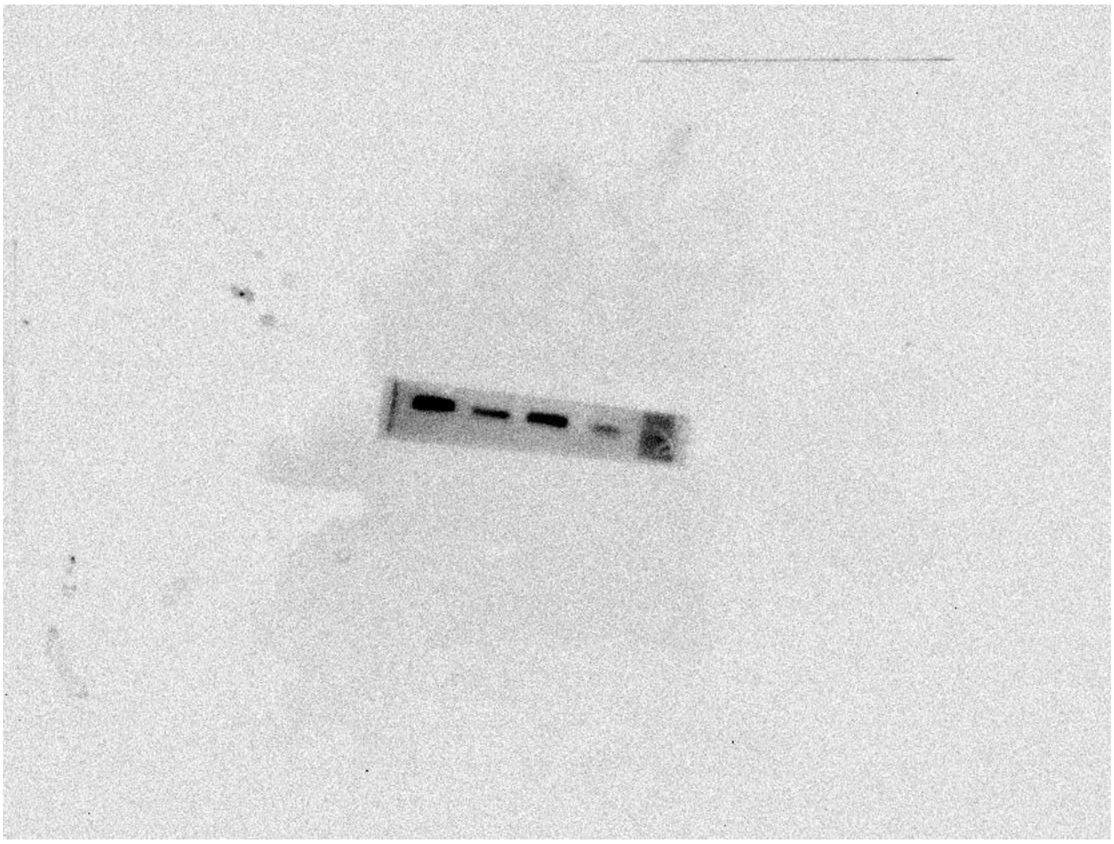

GAPDH

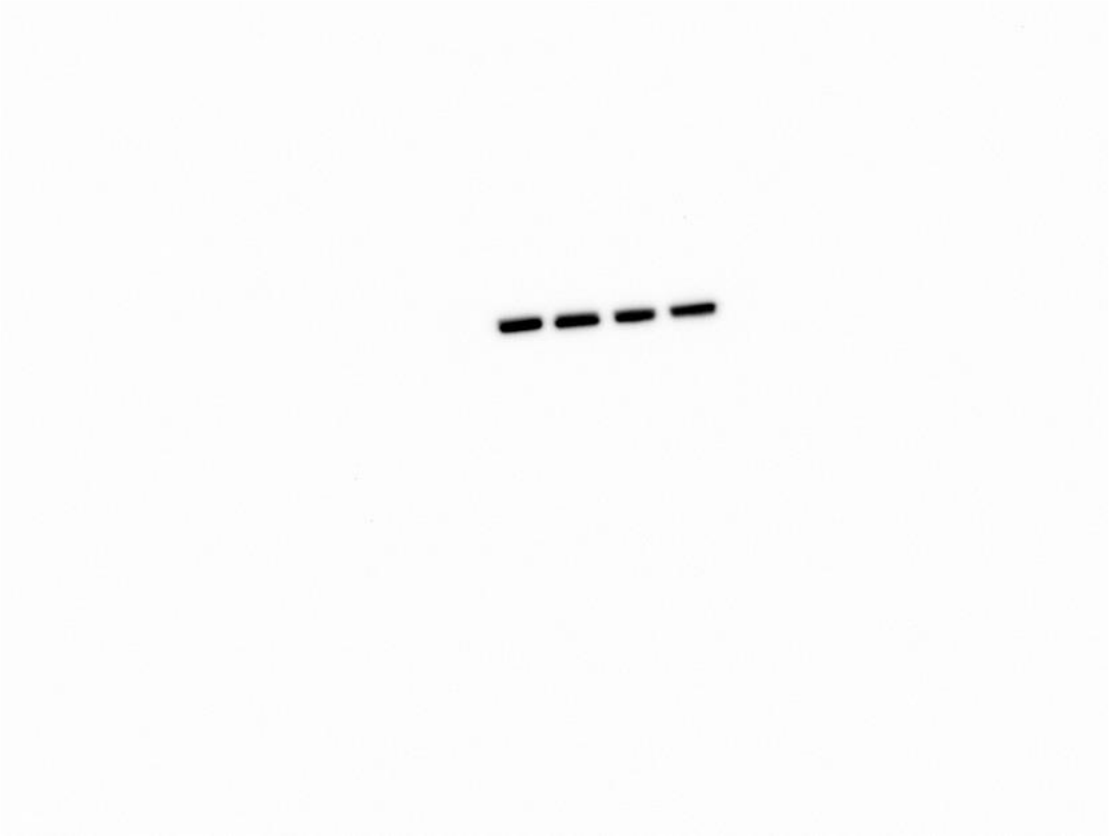

Fig.4 (C)
GALNT7

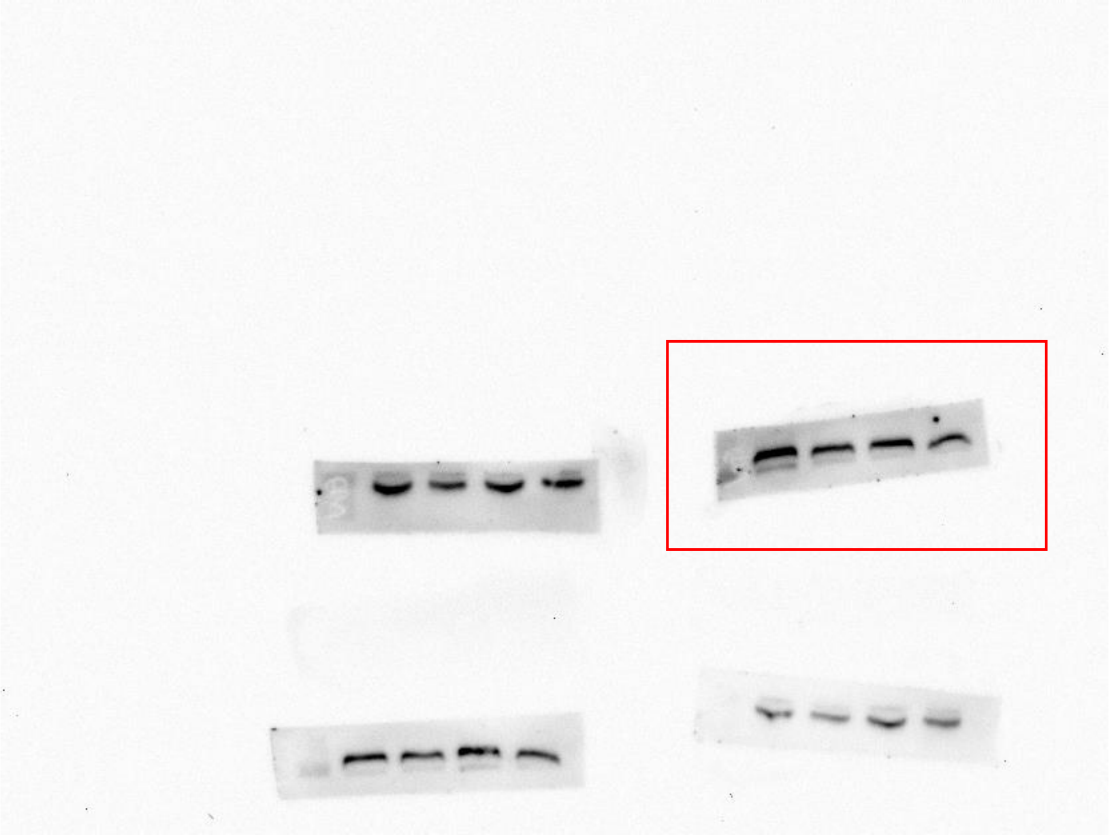

GAPDH

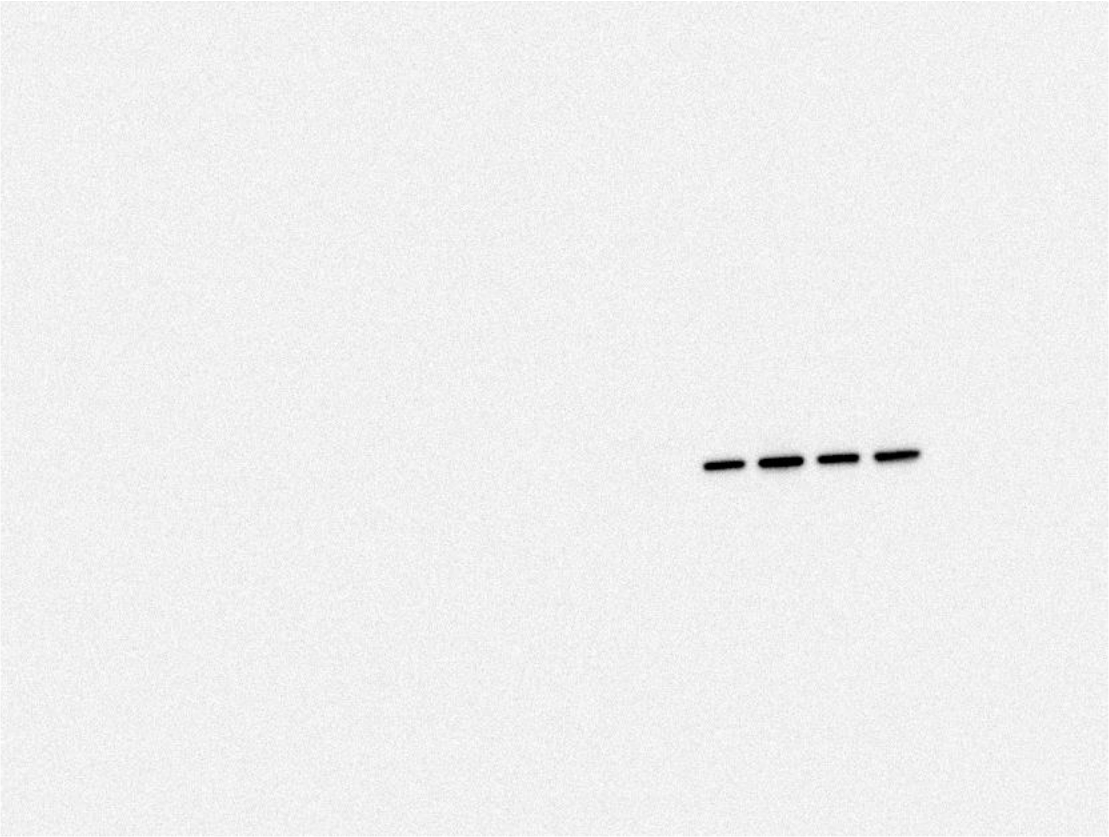

Fig.5 (B)

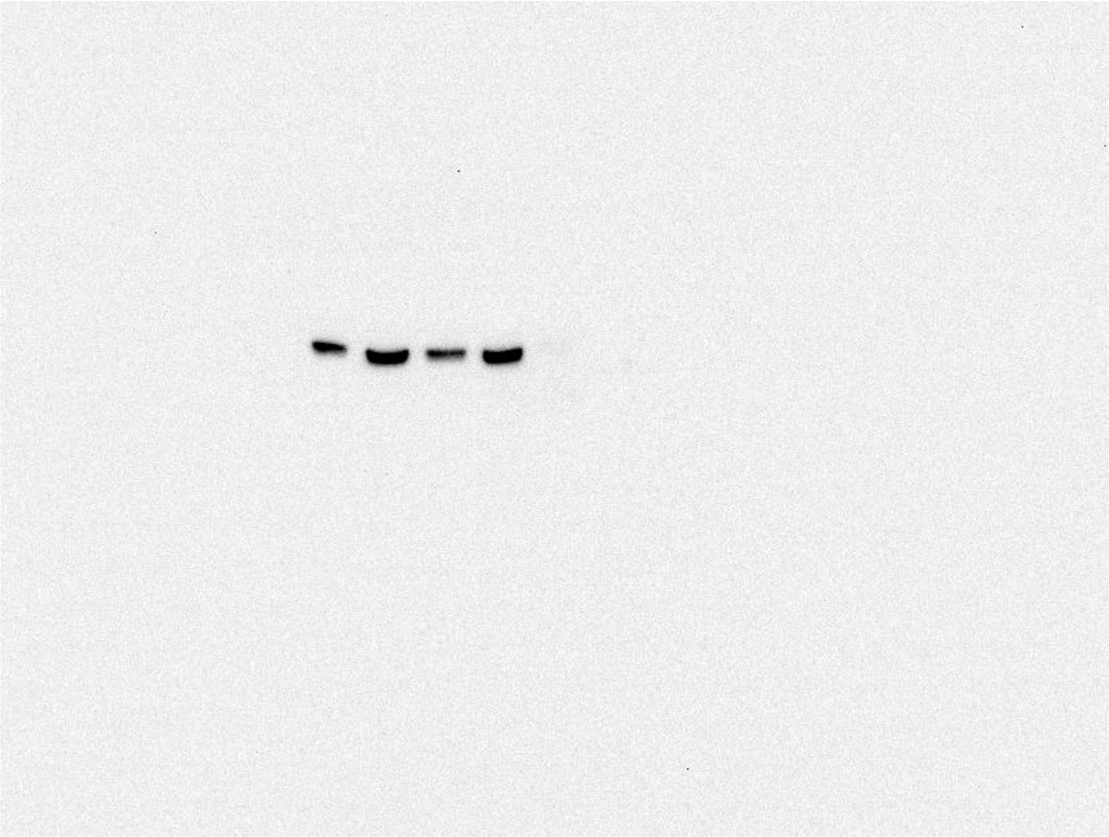

GAPDH

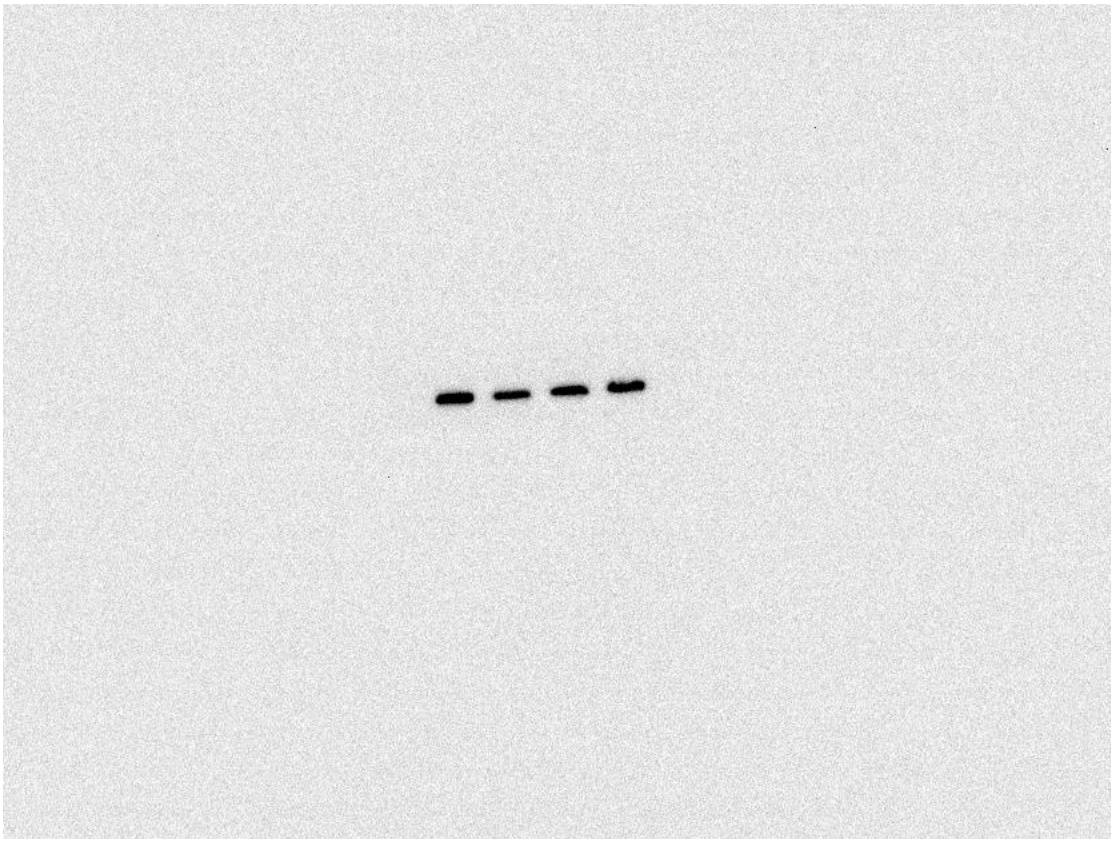

Fig.5(D)

GALNT7(K1)

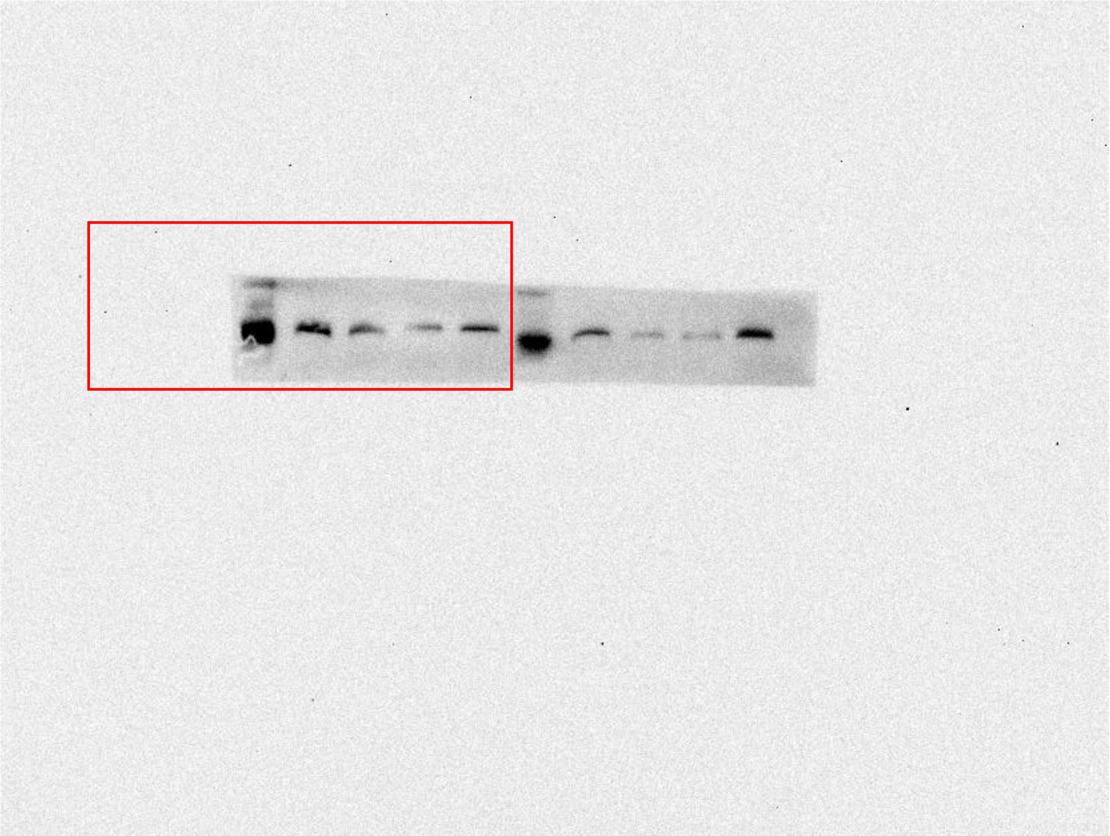

GALNT7(B-CPAP)

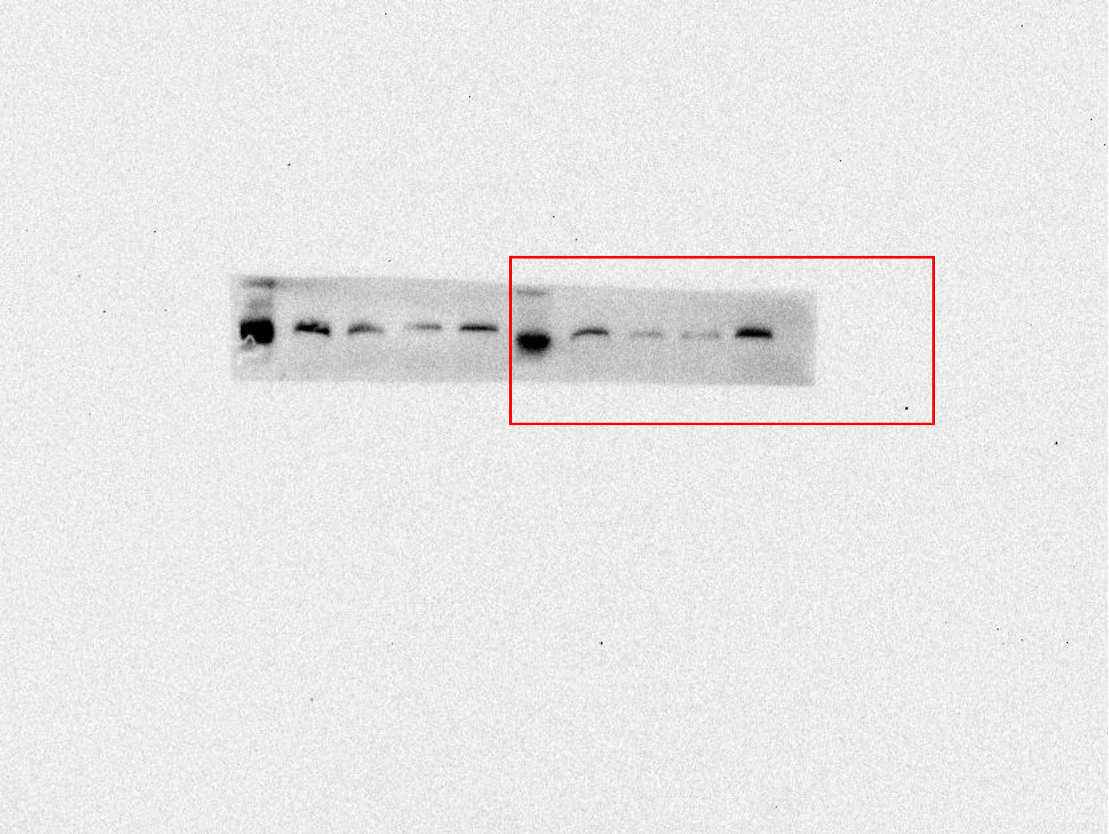

GAPDH(K-1 and B-CPAP)

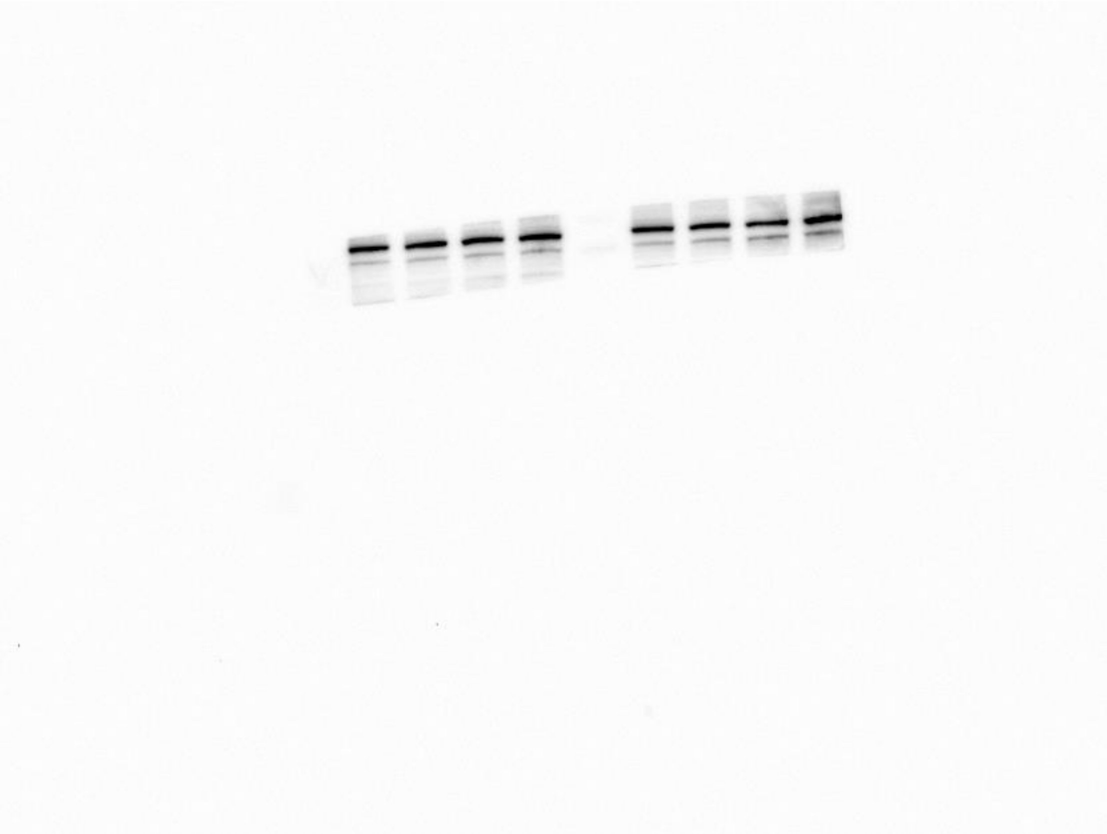

Fig.6 (B)

GALNT7

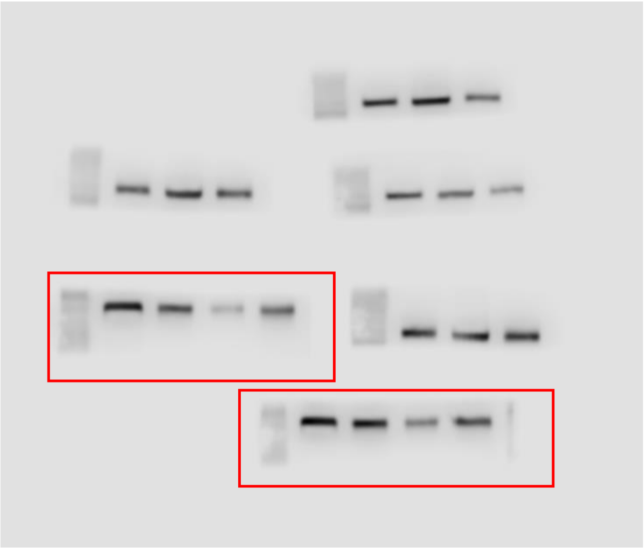

GAPDH

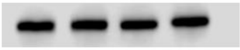

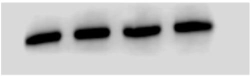

Fig.7 (C)

EGFR

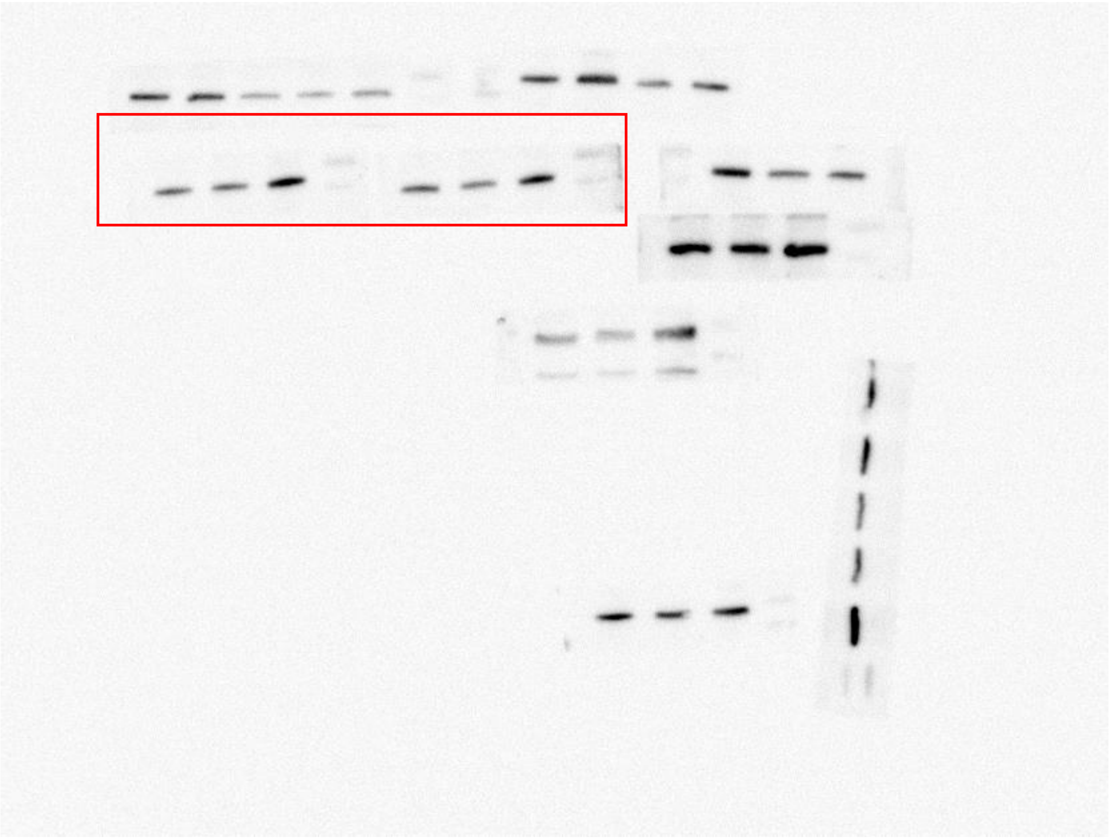

PI3K

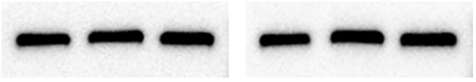

p-PI3K

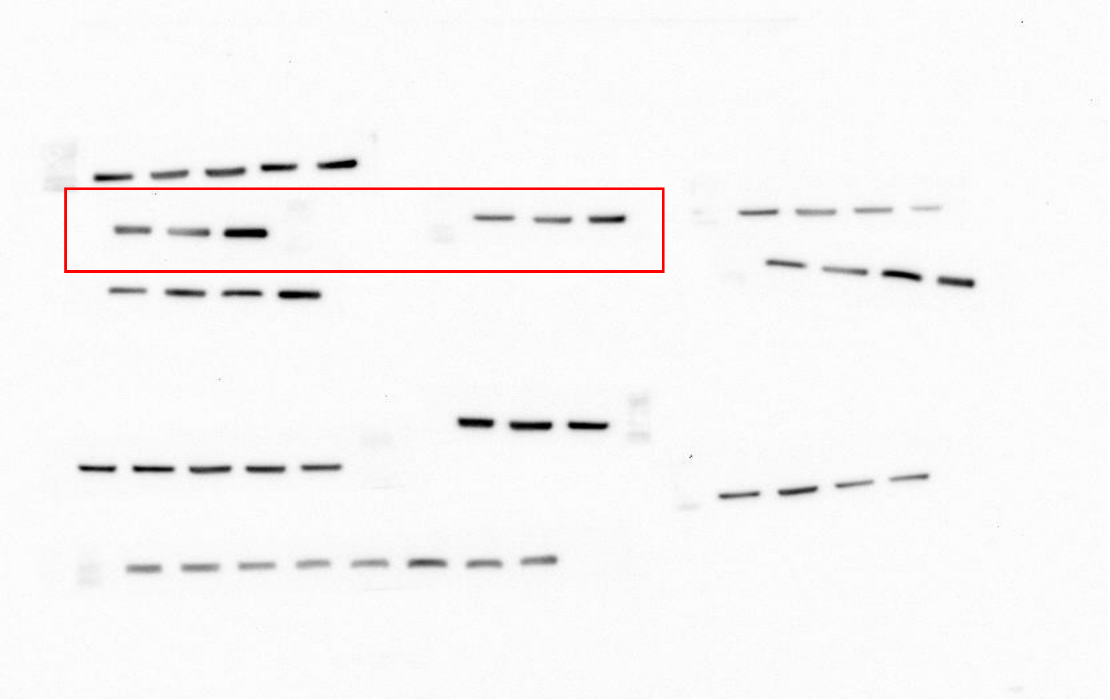

AKT

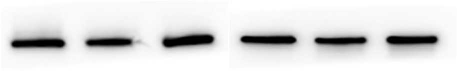

p-AKT

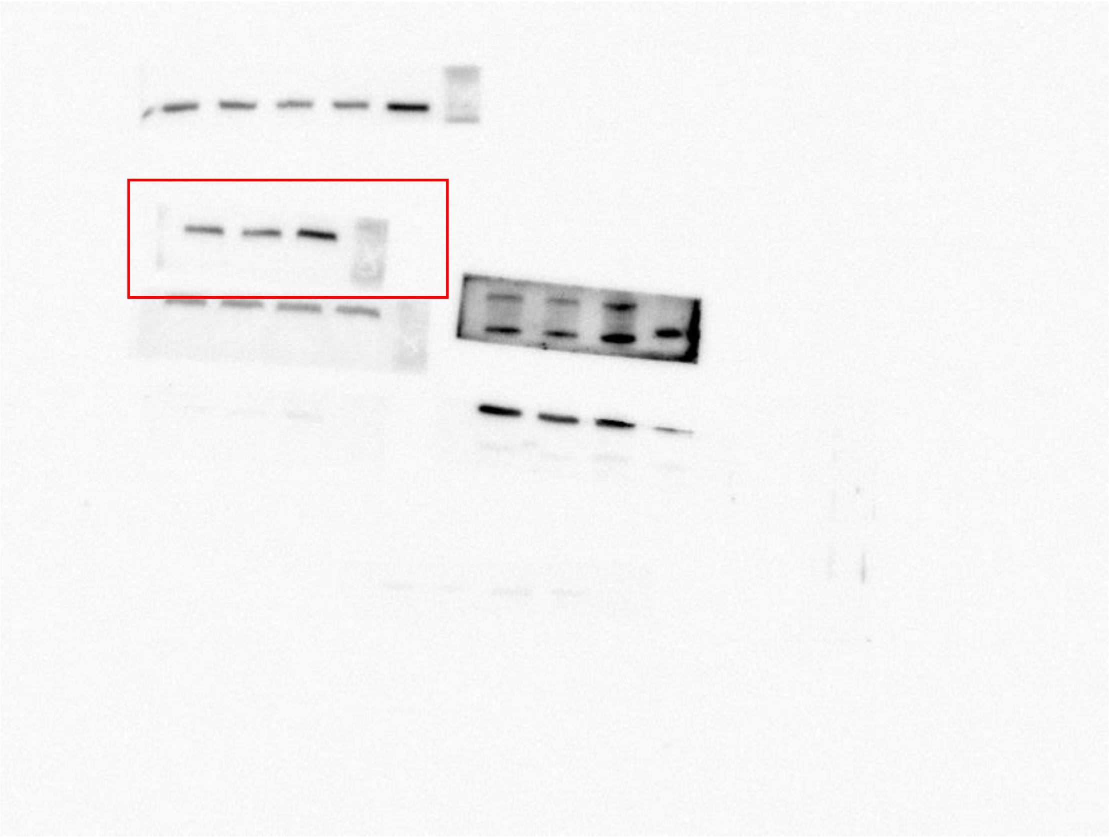

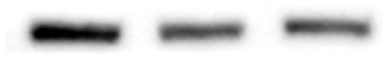

GALNT7

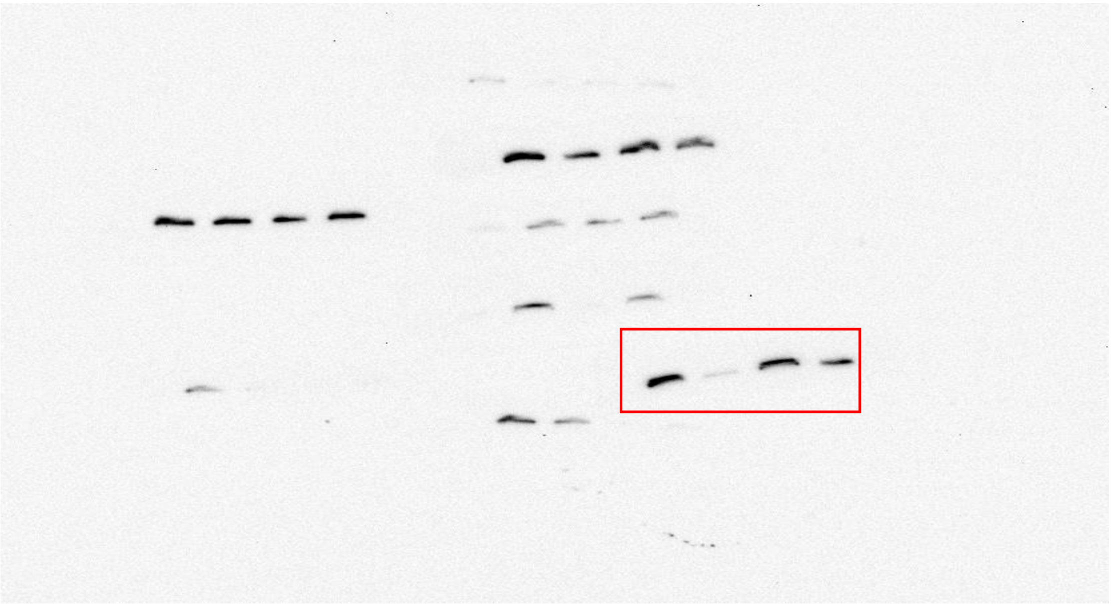

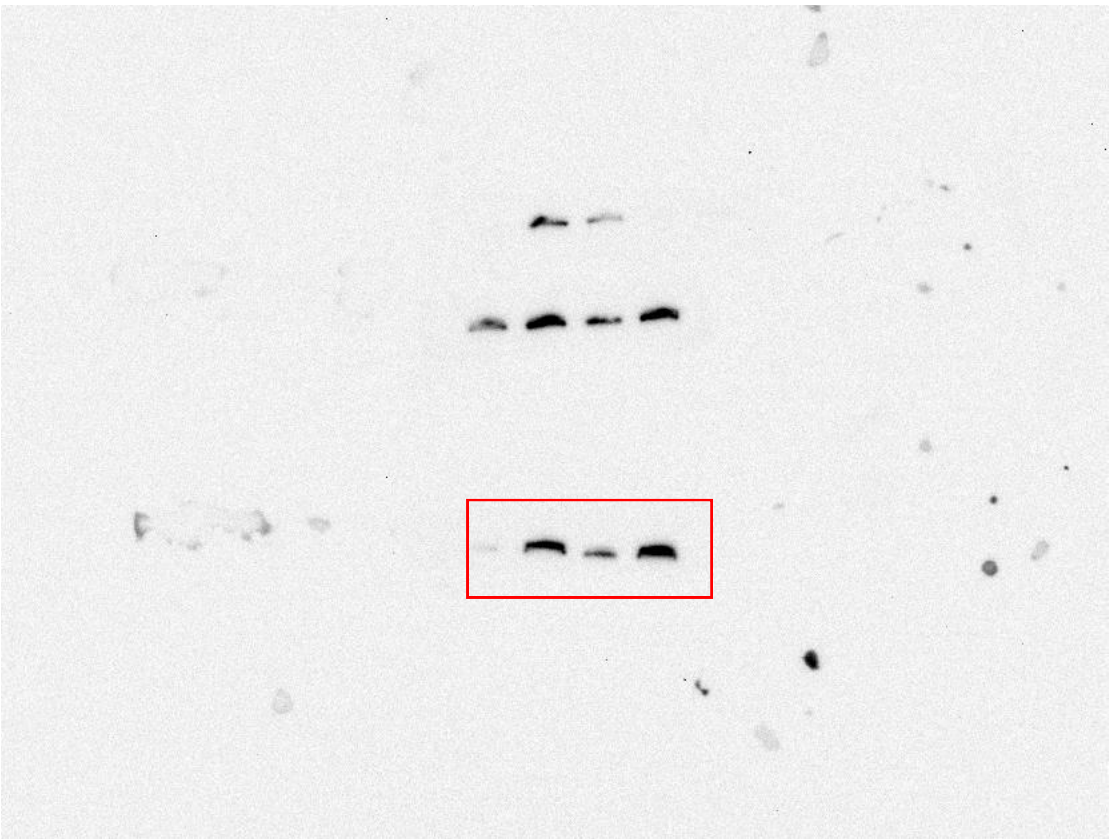

GAPDH

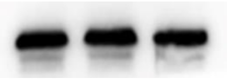

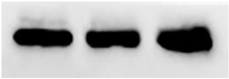

Fig.7 (D)
EGFR

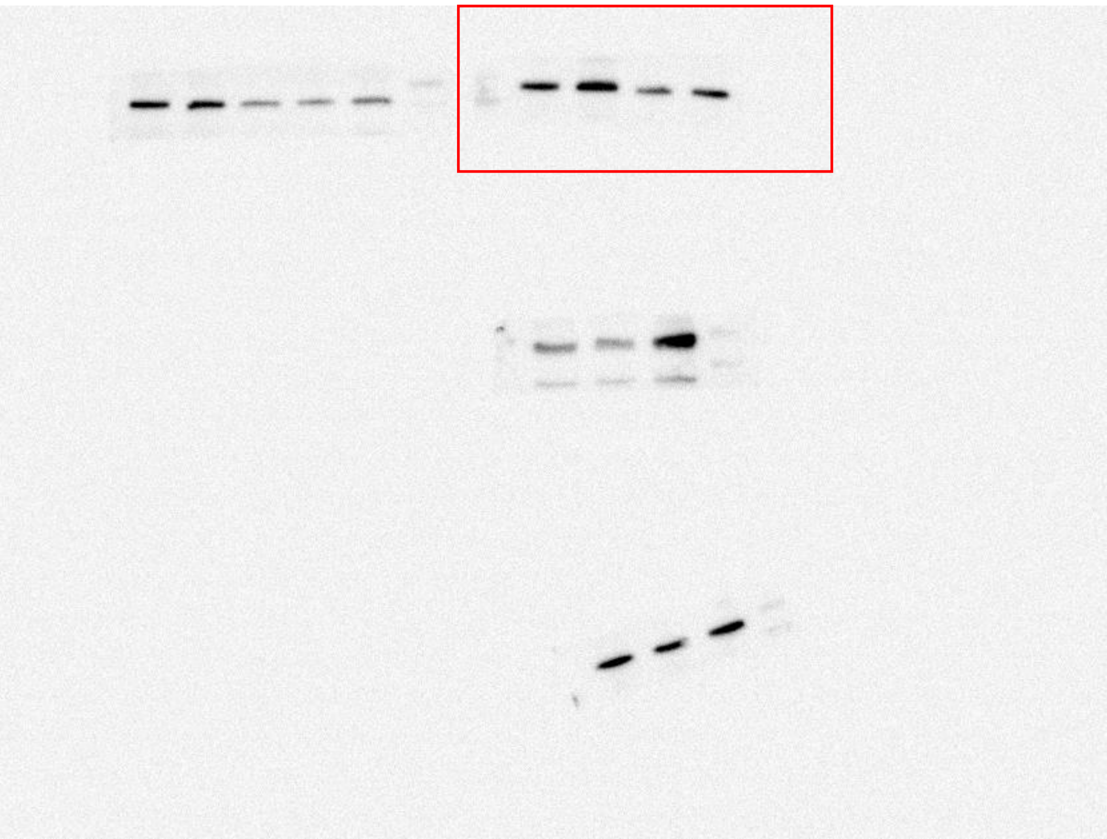

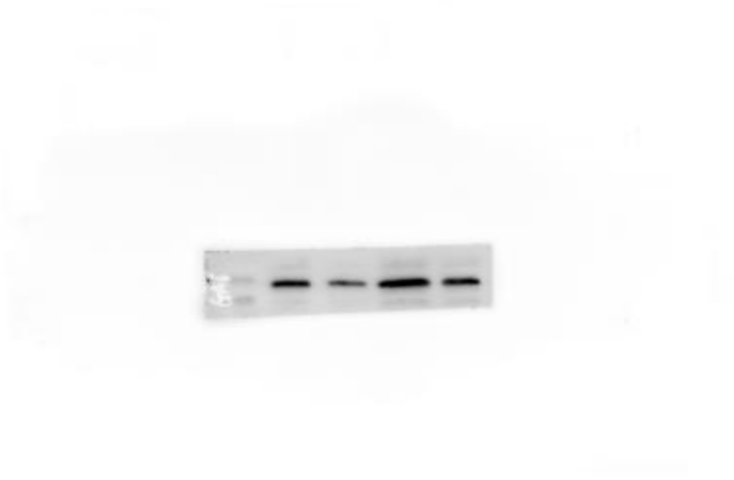

PI3K

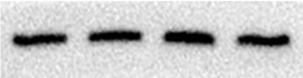

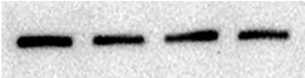

P-PI3K

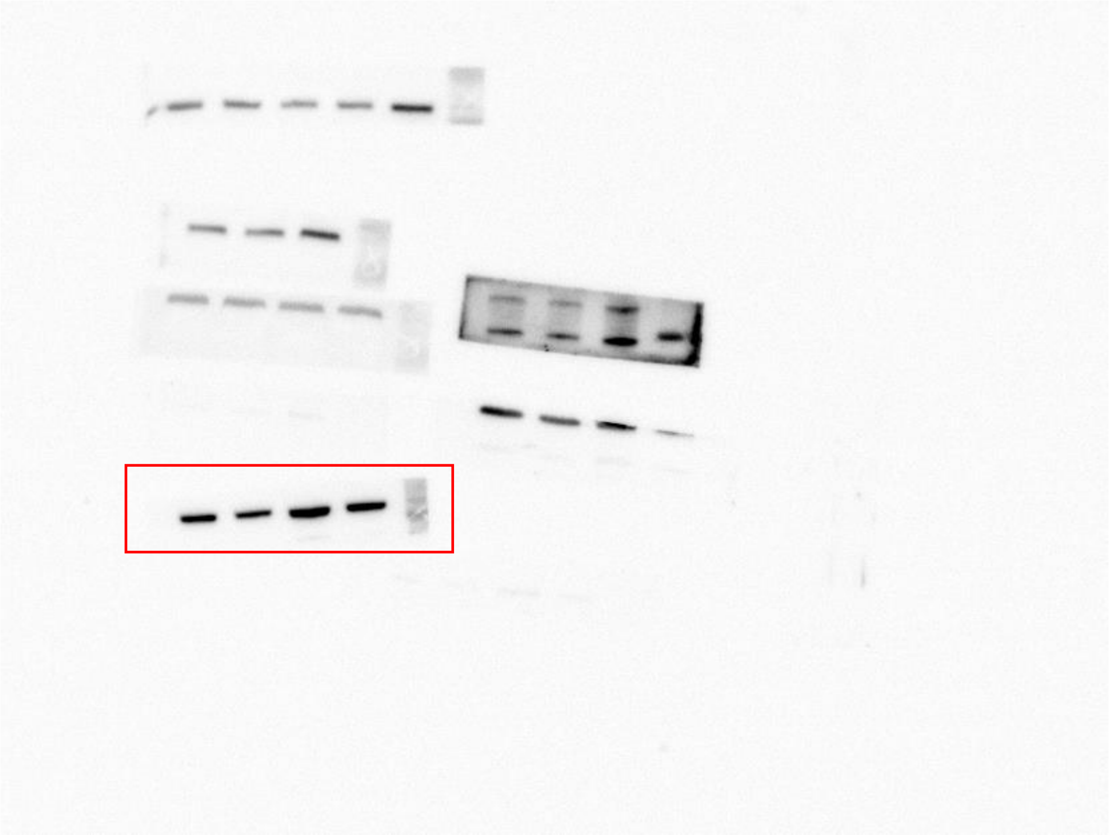

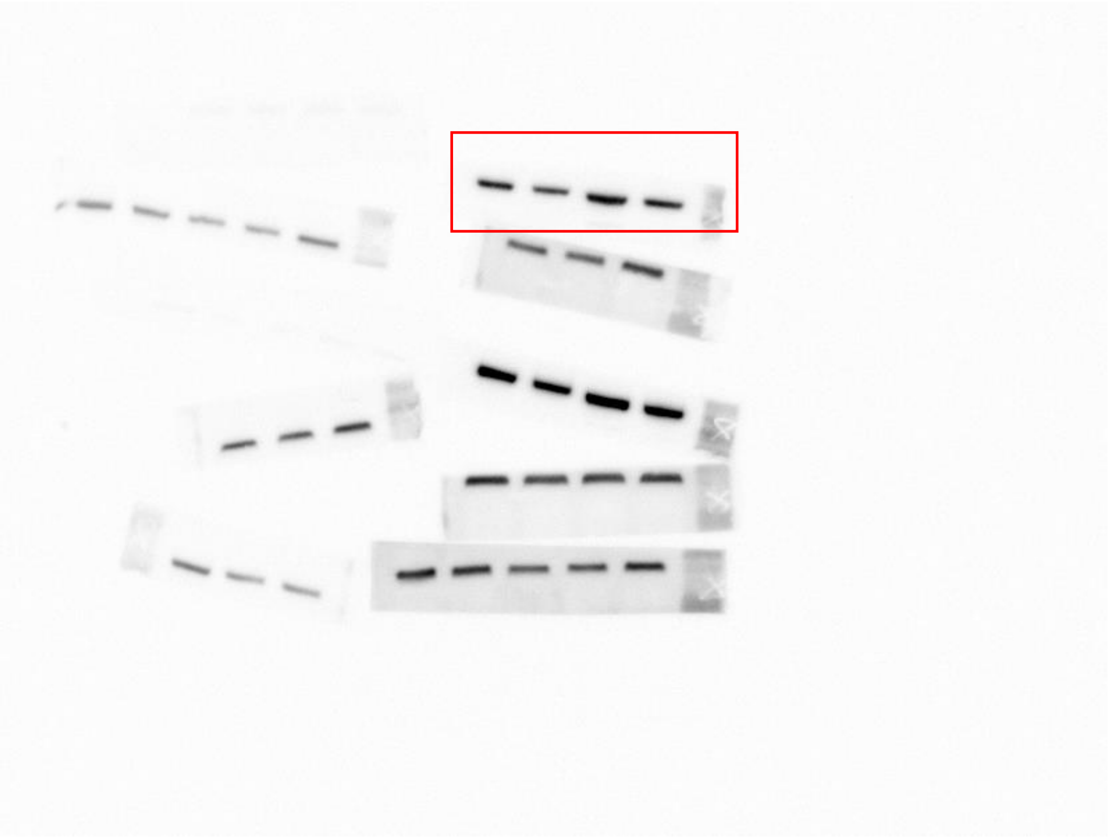

AKT

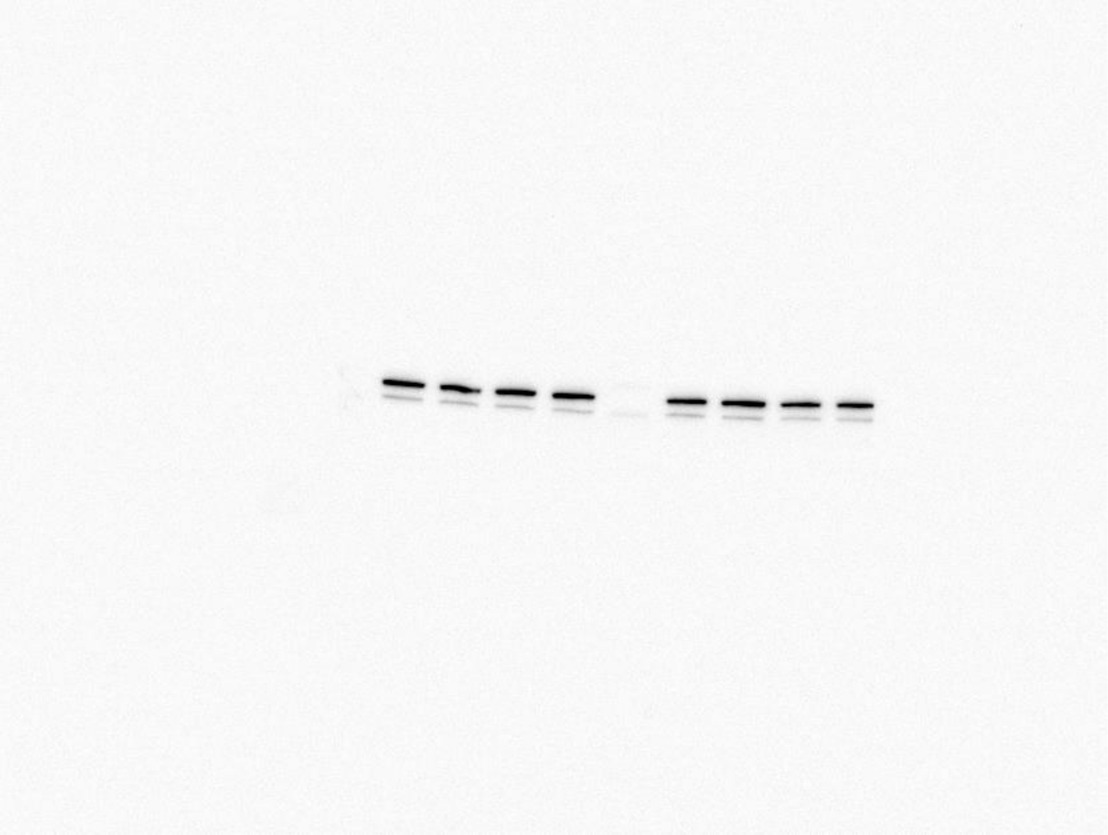

p-AKT

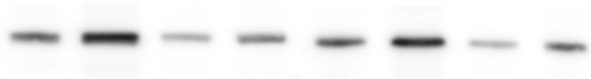

GALNT7

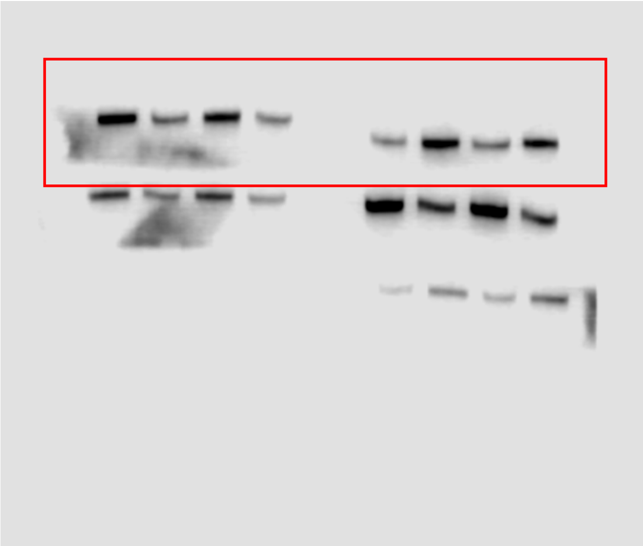

GAPDH

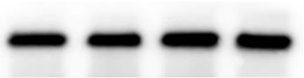

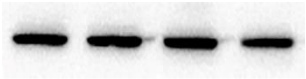

Fig.8(G)

GALNT7

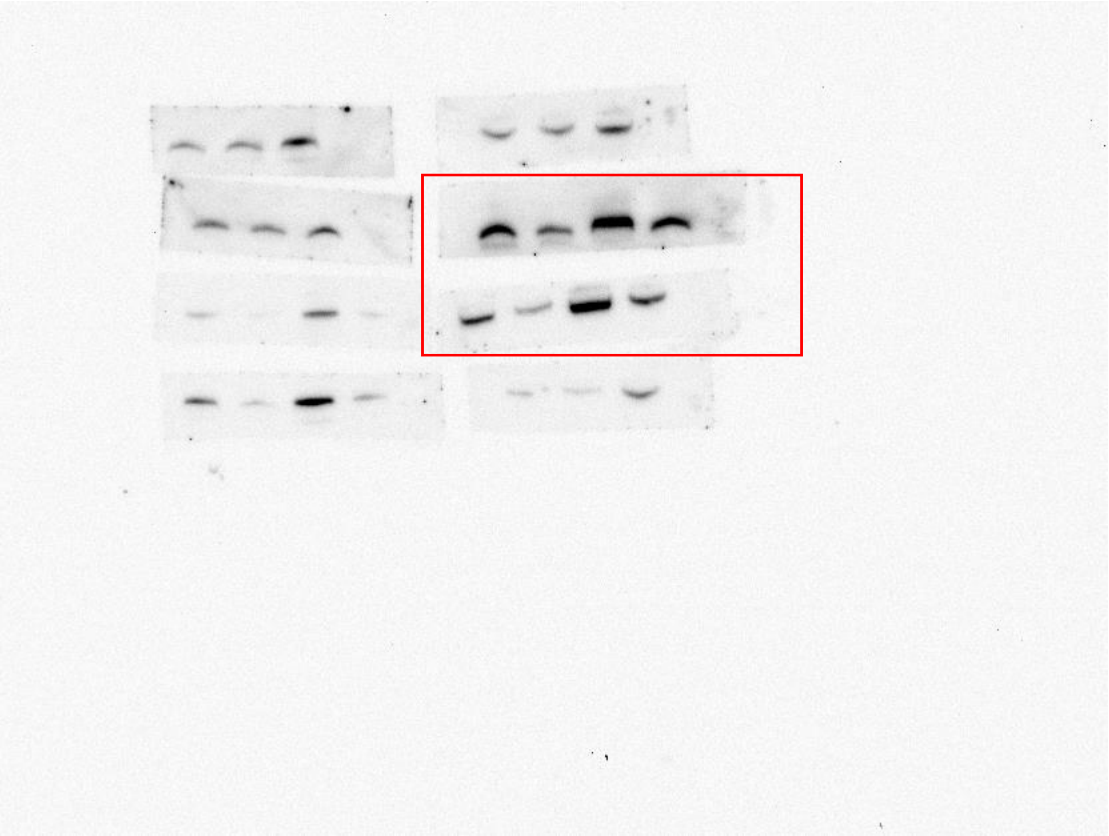

GAPDH

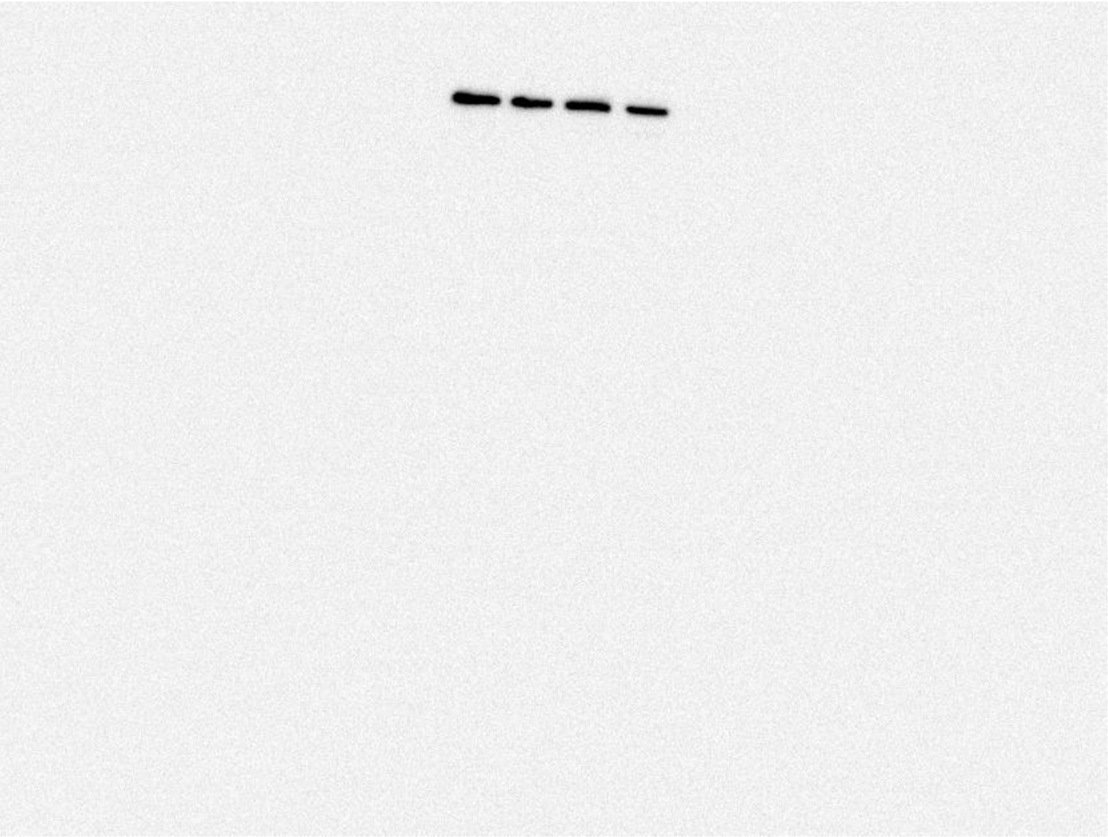

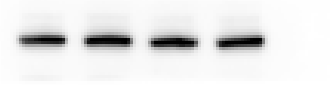

Supplement: Supplementary file 6 — Additional file 6. The original result of western blot is provided as a PDF file. [file 12935_2021_2323_MOESM6_ESM.pdf]
