# Supplementary figures and images for: miR-30b-5p inhibits proliferation, invasion, and migration of papillary thyroid cancer by targeting GALNT7 via the EGFR/PI3K/AKT pathway
Source: Cancer Cell Int. 2021 Nov 24;21:618. doi: 10.1186/s12935-021-02323-x (PMC8611849; doi:10.1186/s12935-021-02323-x)

p-value < 0.0001  
R = 0.49

log2(EGFR TPM)

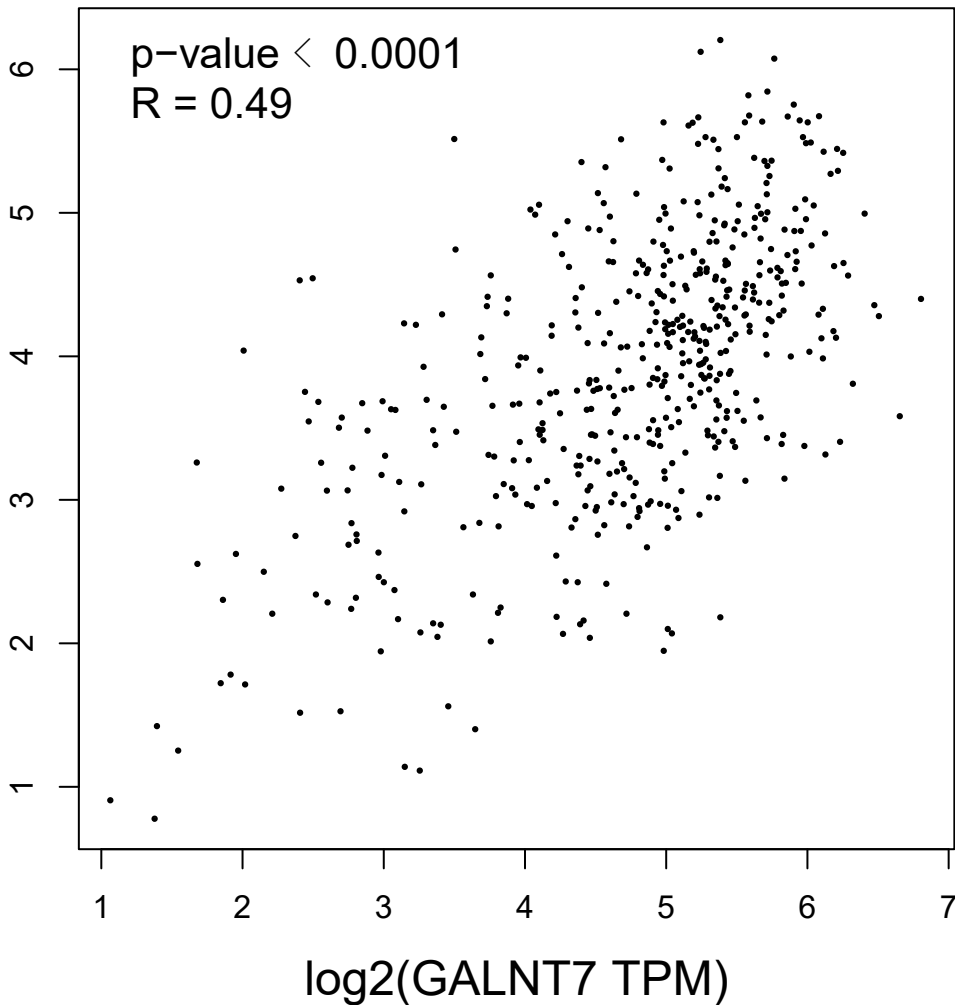

Supplement: Supplementary file 10 — Additional file 10. The results of correlation analysis of GALNT7 and EGFR. [file 12935_2021_2323_MOESM10_ESM.pdf]

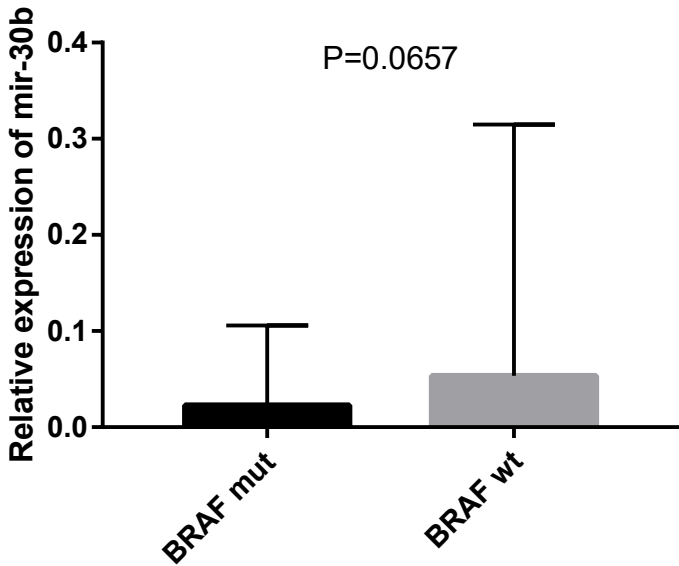

Supplement: Supplementary file 11 — Additional file 11. Differential expression of miR-30b-5p in patients with braf mutant and wild type thyroid carcinoma. [file 12935_2021_2323_MOESM11_ESM.pdf]

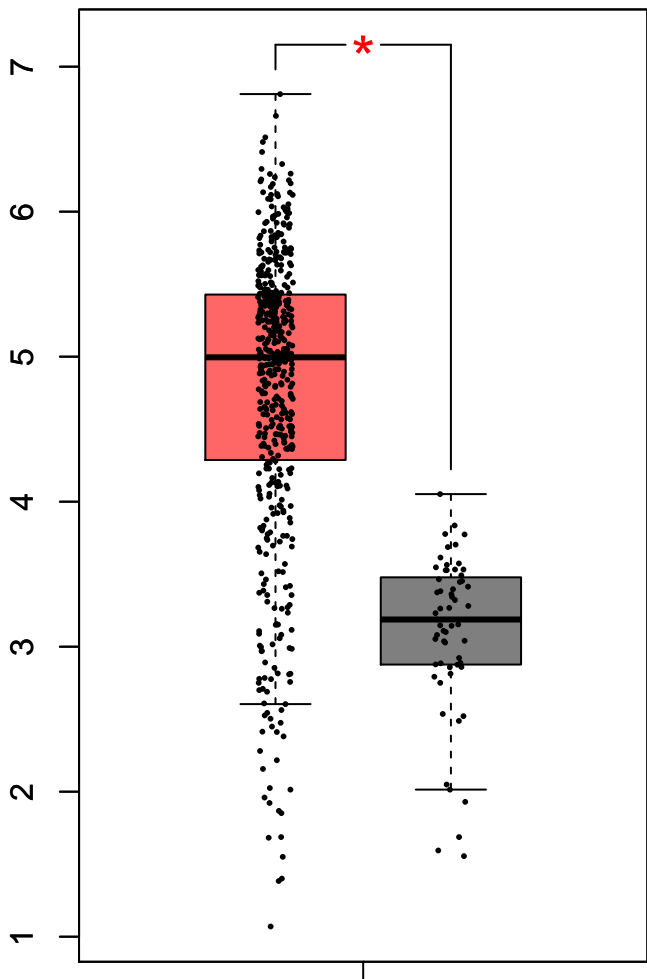

THCA  
(num(T)=512; num(N)=59)

Supplement: Supplementary file 12 — Additional file 12. The expression of GALNT7 in TCGA-THCA datasets. [file 12935_2021_2323_MOESM12_ESM.pdf]
